# Differentiated management of ROS level in tumor and kidney to alleviate Cis-platinum induced acute kidney injury with improved efficacy

**DOI:** 10.1186/s12951-024-02710-2

**Published:** 2024-07-24

**Authors:** Shiqi Zhu, Linlin Huo, Jie Zeng, Rong Chen, Yutong Sun, Mingya Tan, Mengke Fan, Meiling Liu, Jiayi Zhao, Guoming Huang, Yi Wang, Zhibo Xiao, Zhenghuan Zhao

**Affiliations:** 1 College of Basic Medical Sciences, Chongqing Medical University, Chongqing, 400016 China; 2 College of Biological Science and Engineering, Fuzhou University, Fuzhou, 350116 P. R. China; 3 Department of Radiology, Daping Hospital, Army Medical University, Chongqing, 400042 China; 4 Department of Radiology, The First Affiliated Hospital of Chongqing Medical University, Chongqing, 400016 China

**Keywords:** Ruthenium nanoparticles, Multi-enzyme activity, Cisplatin adjuvant, Acute kidney injury, Differentiated management of ROS

## Abstract

Cisplatin (DDP) is a prevalent chemotherapeutic agent used in tumor therapy, yet DDP-induced acute kidney injury (AKI) severely limits its clinical application. Antioxidants as reactive oxygen species (ROS) scavengers can circumvent this adverse effect while leading to the decrease of efficacy to tumor. Herein, we report ultrasmall ruthenium nanoparticles (URNPs) as switchable ROS scavengers/generators to alleviate DDP-induced AKI and improve its therapeutic efficacy. In the physiological environment of the kidney, URNPs mimic multi-enzyme activities, such as superoxide dismutase and catalase, effectively protecting the renal cell and tissue by down-regulating the increased ROS level caused by DDP and alleviating AKI. Specifically, URNPs are oxidized by high levels of H_2_O_2_ in the tumor microenvironment (TME), resulting in the generation of oxygen vacancies and Ru^3+^/Ru^4+^ ions. This unique structure transformation endows URNPs to generate singlet oxygen (^1^O_2_) under laser irradiation and hydroxyl radicals (∙OH) through a Fenton-like reaction in tumor cell and tissue. The simultaneous generation of multifarious ROS effectively improves the efficacy of DDP in vitro and in vivo. This TME-responsive ROS scavenger/generator acts as an adjuvant therapeutic agent to minimize side effects and improve the efficacy of chemotherapy drugs, providing a new avenue to chemotherapy and facilitating clinical tumor therapy.

**Graphical Abstract:**

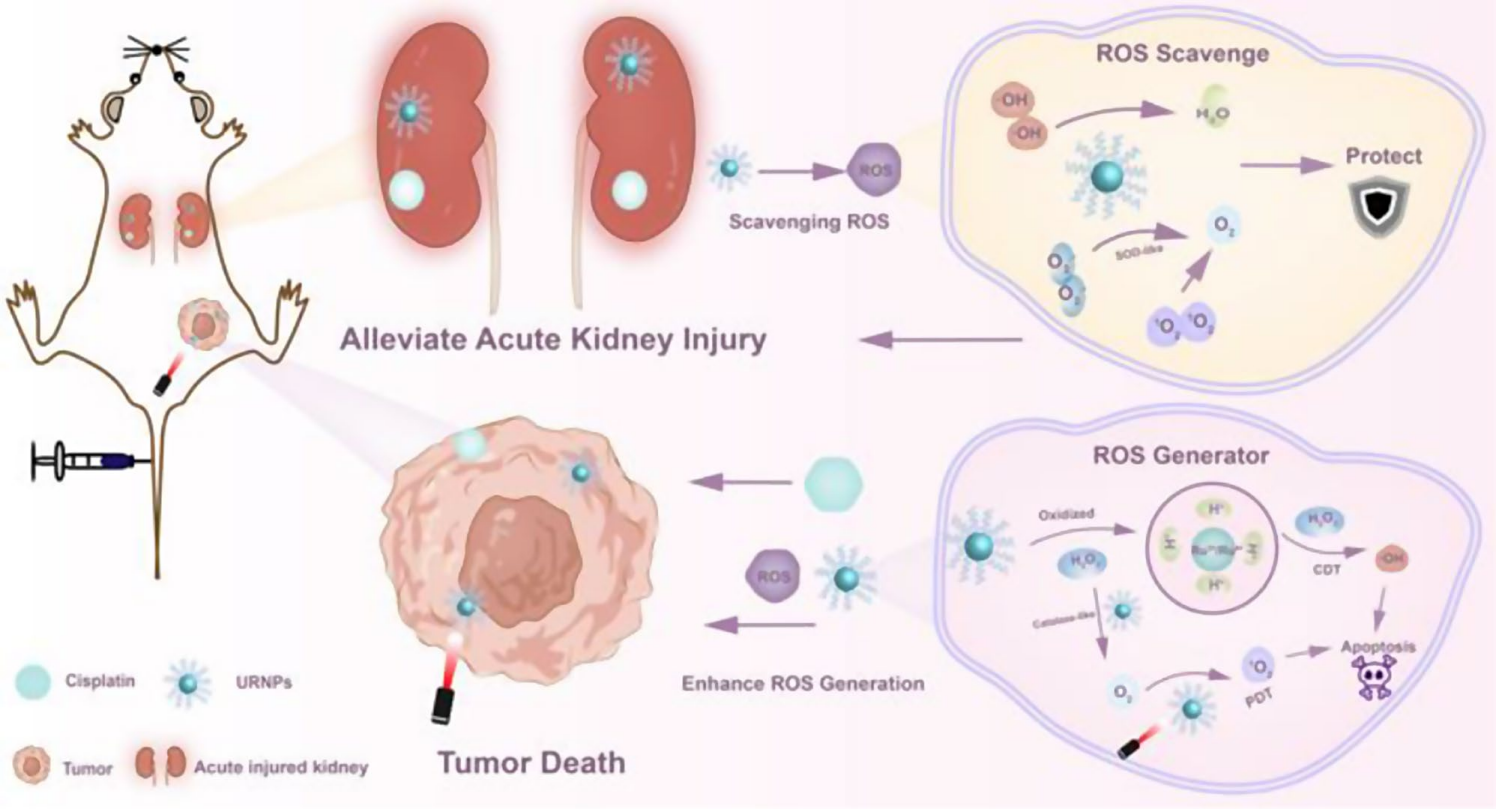

**Supplementary Information:**

The online version contains supplementary material available at 10.1186/s12951-024-02710-2.

## Introduction

Cisplatin (DDP) is a widely used chemotherapy agent for treating solid tumors, such as head, neck, lung, ovarian, and bladder cancers [1, 2]. However, the clinical application of DDP is impeded by severe side effects [3]. DDP-induced acute kidney injury (AKI) is a major limiting factor in DDP based tumor therapy, affecting 30% of DDP receptor patients [4]. Since DDP-induced AKI is found to be cumulative and dose-dependent, dosage reduction or dose discontinuation has been considered as an effective strategy to relieve DDP-induced AKI in clinical [5]. Unfortunately, these strategies come at the cost of chemotherapeutic efficacy, resulting in limited tumor suppression. Excessive reactive oxygen species (ROS) produced by DDP in the kidneys lead to oxidative stress and inflammation, which are associated with AKI [6, 7]. Some natural compounds, such as N-acetylcysteine and vitamin E, have been used as antioxidants to treat DDP-induced AKI [8]. However, its efficacy is highly limited by lacking effective antioxidant efficacy, which hardly fulfills clinical demand. Owing to its high catalytic activity, low cost, and stability, nanozymes have attracted much attention as promising antioxidants [9, 10]. Recently, DNA origami nanostructures [11], black phosphorus nanosheets [12], and copper-based nanoparticles [13] have been used as ROS scavengers to alleviate DDP-induced AKI. Unfortunately, these antioxidants simultaneously decrease oxidative stress at the tumor site and may interfere with tumor progression, leading to metastatic spread and distant metastases [14, 15]. Catalytic tunable ceria nanozyme has been developed to provide a novel strategy to scavenge DDP-induced ROS by modulating the context-dependent [16]. Although this intelligent nanozyme shows negligible interference with the chemotherapeutics of DDP, it is still powerless to increase the efficacy of DDP.

ROS-based tumor treatments, for example, photodynamic therapy (PDT) and chemodynamic therapy (CDT) [17, 18], can overcome the shortcomings of insufficient efficacy and resistance of chemotherapeutic drugs. PDT induces tumor cell death by exciting photosensitizers to produce ROS in the target tumor tissue with the advantages of being non-invasive, highly selective, and low side effects compared to conventional chemotherapy [19]. Simultaneously, some magnetic ions, including Fe [20, 21], Cu [22, 23], and Ru [24], can generate hydroxyl radical (∙OH) in situ to induce apoptosis and inhibit tumor growth through Fenton reaction in the tumor microenvironment. Notably, this method guarantees a degree of safety for normal tissues, as the Fenton reaction or Fenton-like reaction is not activated in physiological environment with neutral and insufficient hydrogen peroxide [25, 26]. Consequently, integrating DDP with ROS-mediated therapies is a promising strategy to enhance its anti-tumor effects [27]. Unfortunately, these strategies are helpless to reduce the ROS level in the kidney caused by DDP-induced AKI.

Differentiated management of ROS level in tumor and kidney could simultaneously alleviate DDP-induced AKI and improve efficacy of DDP. However, it remains a challenge to develop novel adjuvant therapeutic agents that can reduce ROS level in the kidney to alleviate DDP-induced AKI while increase ROS level in tumor to improve efficacy of DDP. Herein, we report ultrasmall ruthenium nanoparticles (URNPs) with tumor microenvironment responsive switchable ROS scavenging/generating activity to alleviate DDP-induced AKI and improve DDP efficacy. In physiological environment of the kidney, URNPs show remarkable mimic superoxide dismutase (SOD), mimic catalase (CAT), and hydroxyl radical scavenging activity to effectively eliminate DDP-induced excess ROS. These distinctive features empower URNPs to not only prevent the occurrence of AKI but also alleviate AKI during DDP treatment in vivo. More importantly, URNPs switch from ROS scavenger to ROS generator in tumor with acidic and overexpressed hydrogen peroxide [28, 29] owing to the valence state variation of Ru and generation of oxygen vacancies. Oxidized URNPs generate sufficient ∙OH via Fenton-like reaction and ^1^O_2_ under 808 nm laser irradiation due to the existence of Ru^3+^ and oxygen vacancy, achieving efficient tumor suppression and significantly increasing the therapeutic efficacy of DDP in vitro and vivo. In conclusion, URNPs display context-related ROS modulation capacity with synergistic toxic potential against cancer while alleviating DDP toxicity, providing new perspectives for the development of an intelligent adjuvant agent to assist DDP tumor therapy.

## Results and discussion

### Synthesis and characterization of ultrasmall ruthenium nanoparticles (URNPs)

**Scheme 1:**
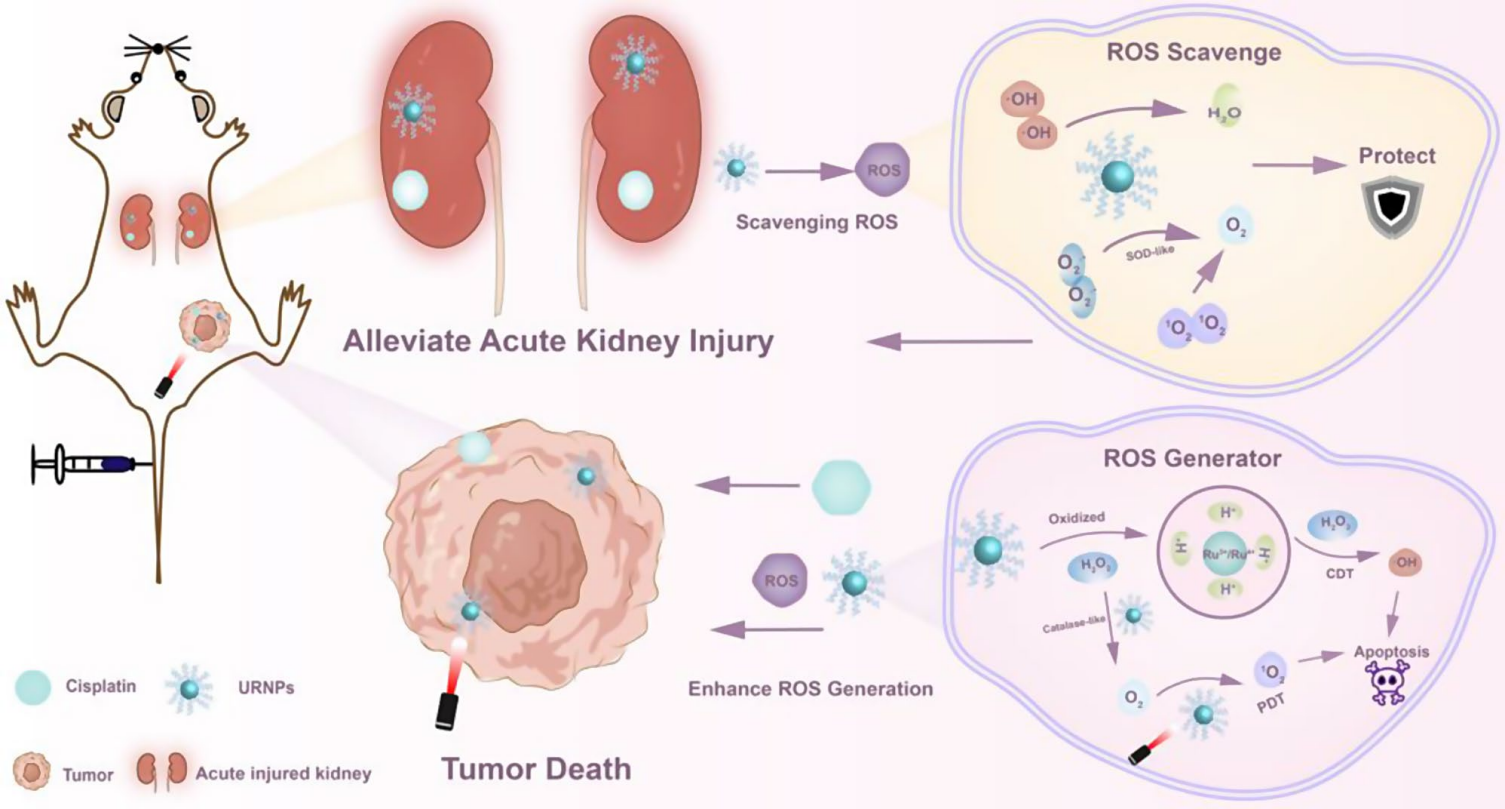
Schematic illustration of URNPs differentially managing ROS level in tumor and kidney. In the physiological environment of the kidney, URNPs mimic multi-enzyme activities to protect the kidney by scavenging ROS induced by DDP. In tumor microenvironments, URNPs selectively act as ROS generators to kill tumors that enhance the efficacy of DDP

**Fig. 1:**
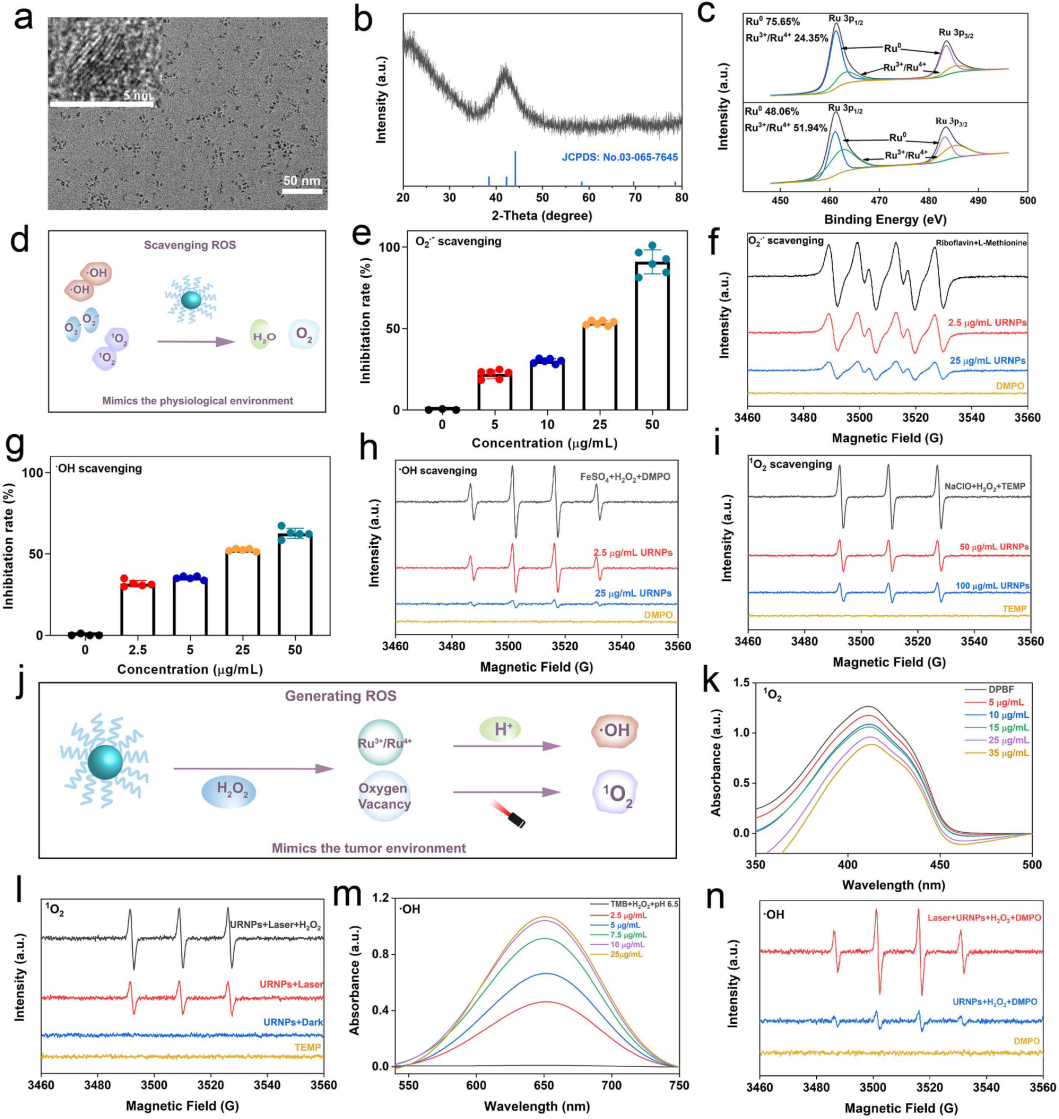
Characterization of URNPs. (**a**) TEM image of URNPs, scale bar is 50 nm. Insert is high-resolution TEM image of URNPs, scale bar is 5 nm. (**b**) XRD patterns of URNPs. (**c**) XPS analyses of URNPs before and after oxidizing by H_2_O_2_. (**d**) Schematic illustration of ROS scavenging capacity of URNPs in a physiological environment. (**e**) UV-Vis spectra of O_2_.^−^ scavenging of URNPs at different concentrations in the pH 7.4 solution. (**f**) ESR spectra analyses of O_2_.^−^ scavenging of URNPs. (**g**) UV-Vis spectra of ·OH scavenging of URNPs at different concentrations in the pH 7.4 solution. (**h**) ESR spectra analyses of ·OH scavenging of URNPs. (**i**) ESR spectra analyses of ^1^O_2_ scavenging of URNPs. (**j**) Schematic illustration of ROS generating of URNPs in the tumor environment. (**k**) UV-Vis spectra of ^1^O_2_ generation level in the pH 6.5 solution containing different concentrations of URNPs, H_2_O_2_, and DPBF under laser irradiation. (**l**) ESR spectra analyses of ^1^O_2_ generation by URNPs in different treatments. (**m**) UV-Vis spectra of ·OH generation level in the pH 6.5 solution containing different concentrations of URNPs, H_2_O_2_, and TMB. (**n**) ESR spectra analyses of ·OH generation level with URNPs in different treatments

Uniform URNPs were synthesized via a thermo-decomposition method. Transmission electron microscopy (TEM) images show that the as-prepared products show spherical morphology with an average diameter of about 2.80 ± 0.50 nm (Fig. 1a and Figure S1). To investigate the crystal structure of URNPs, we performed X-ray diffraction (XRD) analyses. As shown in Fig. 1b, the characteristic peaks that are similar to the typical ruthenium patterns with hexagonal close-packed (hcp) structure (JCPDS No. 03-065-7645) are observed in XRD patterns. According to X-ray photoelectron spectroscopy (XPS) analyses, the valence of ruthenium in URNPs is predominantly in the form of Ru^0^, endowing URNPs with high reducibility to decrease reactive oxygen species (ROS) level caused by Cisplatin (DDP) in kidney. We noticed a little Ru^3+^/Ru^4+^ existence in URNPs, which could be ascribed to partial oxidation owing to its ultrasmall size. Interestingly, the dominant species of Ru change to Ru^3+^/Ru^4+^ratio after H_2_O_2_ treatment (Fig. 1c). Since ruthenium element with high valence has been reported as efficient chemodynamical therapy (CDT) agent in acidic environment [30], these results imply that URNPs may act as CDT agent to improve the efficacy of DDP. In order to transfer URNPs into aqueous solution, 1,2-distearoyl-sn-glycero-3-phosphoethanolamine-N-[amino(polyethylene glycol)-2000] has been successfully modified on its surface. Dynamic light scattering (DLS) measurements identified a hydrodynamic diameter of ~ 8.63 nm and a surface zeta potential of ~ 7.34 mV (Figures S2 and S3), which corresponded to the renal filtration threshold (< 10 nm) for passage through the glomerulus and excretion [31, 32]. Furthermore, the stability of URNPs was investigated by co-incubating them with deionized distilled water (ddH_2_O), phosphate buffered saline (PBS), and complete medium for 0, 24, and 72 h. It appears that URNPs monodisperse in those media with high stability, which is beneficial to long-term circulation in vivo (Figure S4) (Scheme 1).

### ROS scavenging activities of URNPs in neutral physiological environment

As acute kidney injury (AKI) was associated with excessive ROS induced by DDP, we assessed the multi-enzyme activity of URNPs to eliminate ROS in a simulated neutral physiological environment (Fig. 1d). The SOD-like enzymatic activity of URNPs was verified by reacting with superoxide anion (O_2_^·−^) generated by the irradiation of methionine and riboflavin. The absorption of nitrotetrazolium blue chloride (NBT) at 560 nm is significantly reduced by URNPs (Fig. 1e). Remarkably, URNPs with the concentration as low as 50 µg/mL even successfully eliminate more than 91% of the O_2_^·−^. Consistent with the UV-Vis analyses, the electron spin resonance (ESR) spectra confirm the concentration-dependent activity of URNPs as a SOD-like enzyme (Fig. 1f). To explore the scavenging ability of URNPs to hydroxyl radical (·OH), we utilized salicylic acid (SA) as a specific probe to detect the ·OH level with or without URNPs in the classical Fenton reaction system. The characteristic signal of ·OH in solution with URNPs is dramatically lower than that of solution without URNPs. (Fig. 1g). It should note that the elimination efficiency rises with the increase of URNPs concentration, which is further supported by UV-Vis analyses using methylene blue (MB) as probe (Figure S5). Additionally, we notice that the signal intensities of DMPO/·OH decrease with the increase of URNPs concentration by ESR spectra (Fig. 1h). We further verified singlet oxygen (^1^O_2_) elimination capacity of URNPs by ESR. The ESR spectra results indicate that the characteristic peaks of 2,2,6,6-tetramethylpiperidine-1-oxyl (TEMP)/^1^O_2_ at presence of URNPs are obviously lower than that without URNPs, revealing the scavenging activity of URNPs to ^1^O_2_ (Fig. 1i). These results demonstrate that URNPs can act as potential antioxidants to eliminate ROS in a neutral physiological environment based on robust multi-enzyme activity [33].

### ROS Generation in mimic tumor environment

As antitumor adjuvant, ROS generation of URNPs was stimulated in mimic TME (Fig. 1j) [34]. Since ^1^O_2_ has been proven to be generated by photosensitizers containing ruthenium [35, 36], we evaluated the feasibility of URNPs to generate ^1^O_2_ using 1,3-diphenylisobenzofuran (DPBF) as probe. We noticed that the signal intensity of DPBF decreased at presence of URNPs under 808 nm laser irradiation, while no apparent change could be observed in the group without URNPs (Figure S6). Besides, these results provide direct evidence of URNPs-mediated ^1^O_2_ generation in concentration-dependent manner (Fig. 1k). Excitingly, the signal intensity of TEMP/^1^O_2_ increases with the introducing of H_2_O_2_ in ESR spectra (Fig. 1l). This phenomenon could be ascribed to the CAT-like activity of URNPs, which catalyze H_2_O_2_ into O_2_ and increase the source of ^1^O_2_ (Figure S7) [37]. To understand the mechanism of ^1^O_2_ generation by URNPs, we analyzed URNPs by ESR spectra. URNPs exhibit a sharp single electron peak, suggesting the existence of oxygen vacancies (OVs) defects in URNPs (Figure S8). The OVs have been proven to provide electron trapping sites to transfer electrons to O_2_ under near-infrared (NIR) laser irradiation [38, 39]. We have previously proved that URNPs could be oxidized by H_2_O_2_ and generate Ru^3+^/^4+^, which have been considered CDT agents. The capacity of the URNPs to generate ·OH was tested using 3,3’,5,5’ -tetramethylbenzidine (TMB) as indicator. It appears that ·OH can be produced even at a low concentration of URNPs (2.5 µg/mL) in the presence of H_2_O_2_ under an acidic environment, as indicated by the solution changing from colorless to blue (Fig. 1m and Figure S9). However, we did not observe apparent signal change of TMB with H_2_O_2_ at pH 7.4 (Figure S10). ESR spectra analyses further confirm URNPs generate numerous ·OH in mimic tumor microenvironment (TME), especially under NIR laser irradiation (Fig. 1n). Notably, the ·OH generation efficiency of URNPs is remarkably increased under laser irradiation due to the good photothermal conversion capacity, which increases the temperature and Fenton-like activity (Figure S11) [40]. The simultaneously generation of toxic ^1^O_2_ and ·OH in mimic TME ensure URNPs to assist DDP to kill tumor cells and improve the efficacy of DDP.

### Protective effect to normal renal cells

**Fig. 2:**
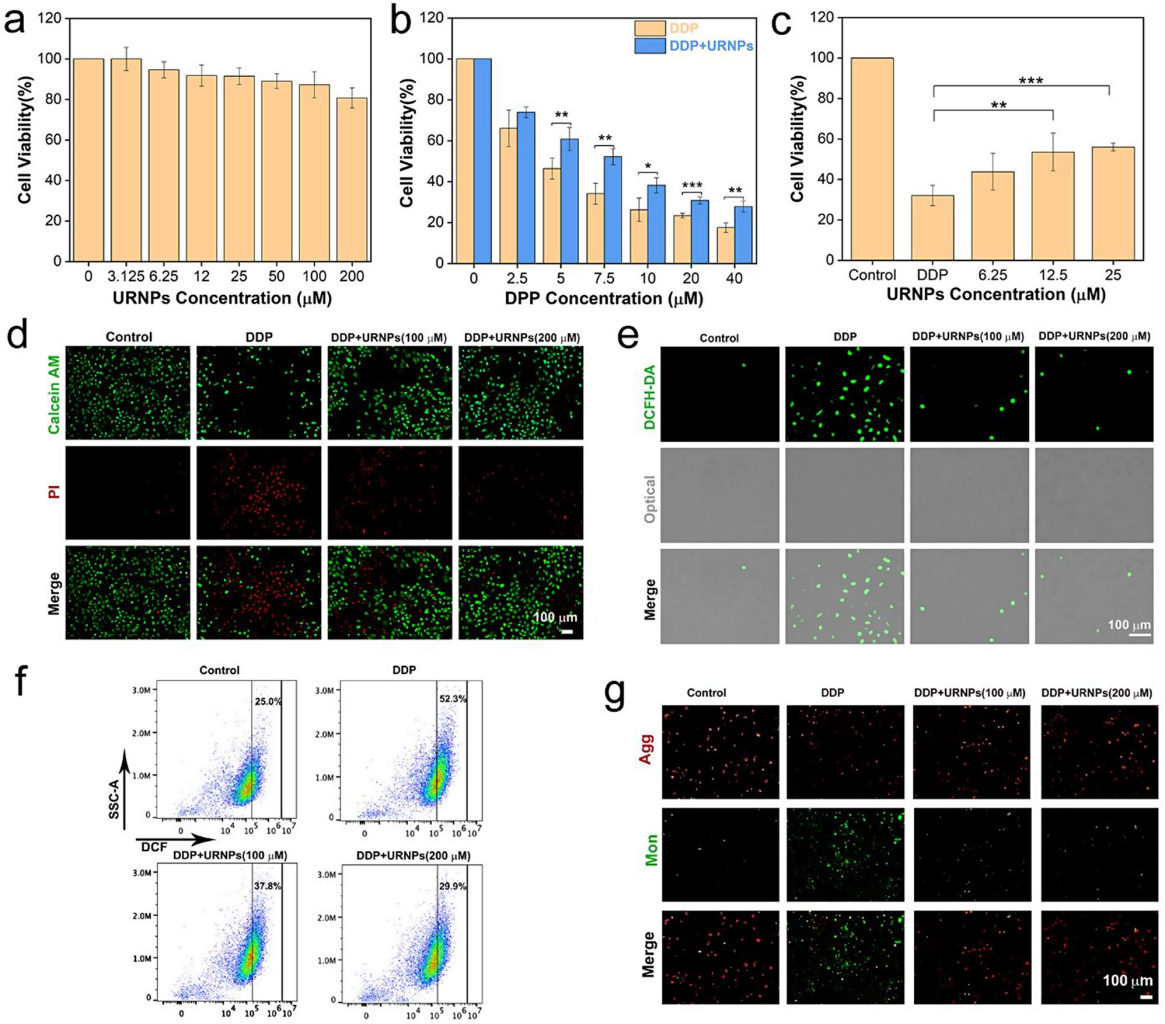
Protective effect on normal renal cells. (**a**) The cell viability of HK-2 cells after treatment with different concentrations of URNPs. (**b**) The cell viability of HK-2 cells after treatment with 100 µM URNPs and different concentrations of DDP. P_(5 µM)_ = 0.0092, P_(7.5 µM)_ = 0.0014, P_(10 µM)_ = 0.0124, P_(20 µM)_ = 0.0004, P_(40 μM)_ = 0.0011.(**c**) The cell viability of HK-2 cells after treatment with 7.5 µM DDP and different concentrations of URNPs. P_(12.5 µM)_ = 0.0014, P_(25 µM)_ = 0.0004. (**d**) Live/dead cell staining tests of HK-2 cells after treatment with 7.5 µM DDP and different concentrations of URNPs. Red, dead cells; green, live cells. Scale bar is 100 μm. (**e**) Fluorescence images of HK-2 cells after different treatments to monitor ROS by using DCFH-DA as an indicator. Scale bar is 100 μm. (**f**) The DCF fluorescence intensity is quantified via flow cytometry. (**g**) Fluorescence images of HK-2 cells after different treatments to detect mitochondrial membrane potential measurement by JC-1 probes. Scale bar is 100 μm. (The two groups were analyzed using Student’s t-test; between three or more groups using one-way ANOVA with multiple comparisons test. *n* = 4, mean ± SD, *p < 0.05, **p < 0.01, and ***p < 0.001)

Renal tubules are typical targets for DDP-induced nephrotoxicity and susceptible to damage by oxidative stress [41]. Consequently, HK-2 cells (human renal tubular epithelial cells) were chosen as model to investigate the protective effects of URNPs on normal renal cells. Before investigating the protective effect, we analyzed the cytotoxicity of URNPs to HK-2 cells by cell-counting-kit-8 (CCK-8) assay. Of note, there is no apparent effect of URNPs on the viability of HK-2 cells (Fig. 2a). The high biocompatibility motivates us to systematically assess the protective effect of URNPs on HK-2 cells. We observe notable decrease in HK-2 cell survival with incubation of DDP. Interestingly, the viability of HK-2 cells treated by DDP significantly increased after introducing URNPs (Fig. 2b). In addition, the viability increase with the rise of URNPs concentration (Fig. 2c). These results clearly reveal the protective effect of URNPs on HK-2 cells. We further used double staining of calcein acetoxymethyl ester (calcein-AM) and propidium iodide (PI) to differentiate dead from live cells. It appears that DDP treated cell show evident red fluorescence, indicating mass HK-2 cells death. Excitingly, the introducing of URNPs lead to dramatic decrease of dead cells (red fluorescence) while increase of live cells (green fluorescence) (Fig. 2d). These results are highly consistent with the CCK-8 assay analyses, demonstrating that URNPs could alleviate DDP-induced cytotoxicity in normal renal cells. Since DDP toxicity has been proven to be positively correlated with oxidative stress, we investigated the effect of URNPs on ROS level in DDP treated HK-2 cells using 2, 7-Dichlorodihydrofluorescein diacetate (DCFH-DA) as probe. It appears that the cells treated with DDP show strong green fluorescence in fluorescence imaging, indicating that DDP increases the ROS level in HK-2 cells (Fig. 2e). Notably, the fluorescence intensity exhibits dramatic decrease in the presence of URNPs owing to its ROS scavenging activity in HK-2 cells (Figure S12). To quantify the fluorescence change, we analyzed the fluorescence of cells treated with different conditions through flow cytometry. It appears that the fluorescence intensity of DCF in the cells treated by both DDP and URNPs is significantly lower than that in the cells treated by DDP, demonstrating that URNPs can reduce the oxidative stress of normal renal cells treated by DDP and effectively protect normal renal cells during DDP treatment (Fig. 2f). Mitochondrial dysfunction caused by high ROS levels is one of the major causes of DDP-induced nephrotoxicity, resulting in a decrease in mitochondrial membrane potential (MMP). Therefore, 5,5,6,6-tetrachloro-1,1,3,3-tetraethyl-imidacarbocyanine iodide (JC-1) staining was used to detect mitochondrial membrane potential to further determine whether URNPs suppress DDP-induced cytotoxicity. The strongest green fluorescence is observed in the cells treated by DDP, while we note significant increase in red fluorescence (aggregate) and decrease in green fluorescent (monomer) after introducing URNPs (Fig. 2g). These results suggest that URNPs protect the mitochondria from ROS damage in normal renal cells.

### Improving the efficacy of DDP to tumor cells

**Fig. 3:**
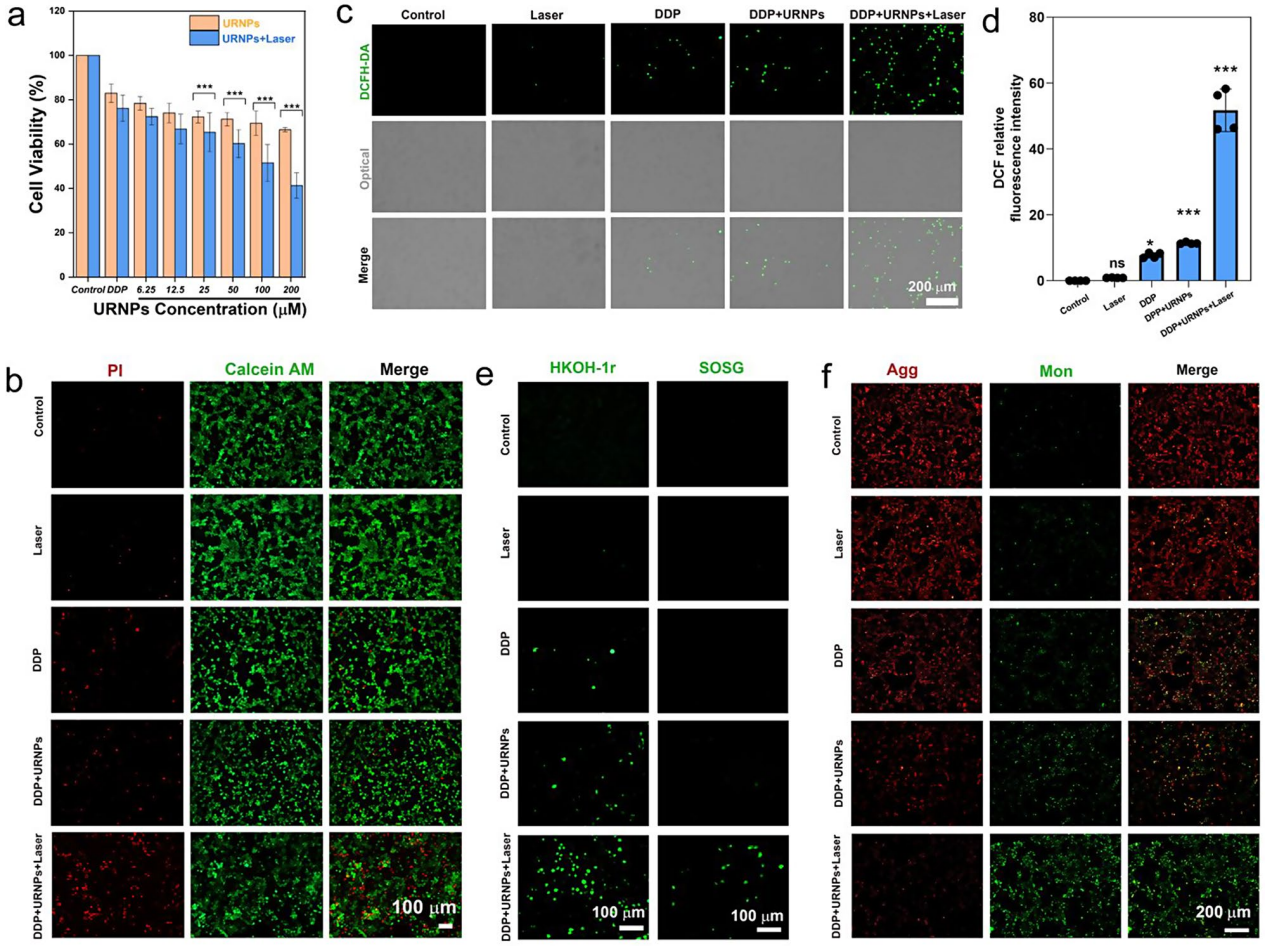
URNPs improve the efficacy of DDP to tumor cells. (**a**) The cell viability of 4T1 cells after treatment by 5 µM DDP and different concentrations of URNPs with or without laser irradiation. P_(25 µM)_;P_(50 µM)_;P_(100 µM)_;P_(200 µM)_ < 0.001. (**b**) Live/dead cell staining tests of 4T1 cells treated by saline, Laser, DDP, DDP + URNPs, and DDP + URNPs + Laser. Red, dead cells; green, live cells. Scale bar is 100 μm. (**c**) Fluorescence images of cells under different treatments to monitor ROS by using DCFH-DA as an indicator. Scale bar is 200 μm. (**d**) Quantitative DCF fluorescence intensity analyses of ROS generation. P_(Laser)_ = 0.9915, P_(DDP)_ = 0.014, P_(DDP + URNPs)_ = 0.0005, P_(DDP + URNPs +Laser)_ < 0.0001. (**e**) The intracellular ·OH and ^1^O_2_ were detected by HKON-1r and SOSG probes as fluorescence probes under different treatments. Scale bar is 100 μm. (**f**) Fluorescence images of cells under different treatments to monitor mitochondrial membrane potential measurement by JC-1 probes. Scale bar is 200 μm. (The two groups were analyzed using Student’s t-test; between three or more groups using one-way ANOVA with multiple comparisons test. *n* = 4, mean ± SD, *p < 0.05, **p < 0.01, and ***p < 0.001)

**Fig. 4:**
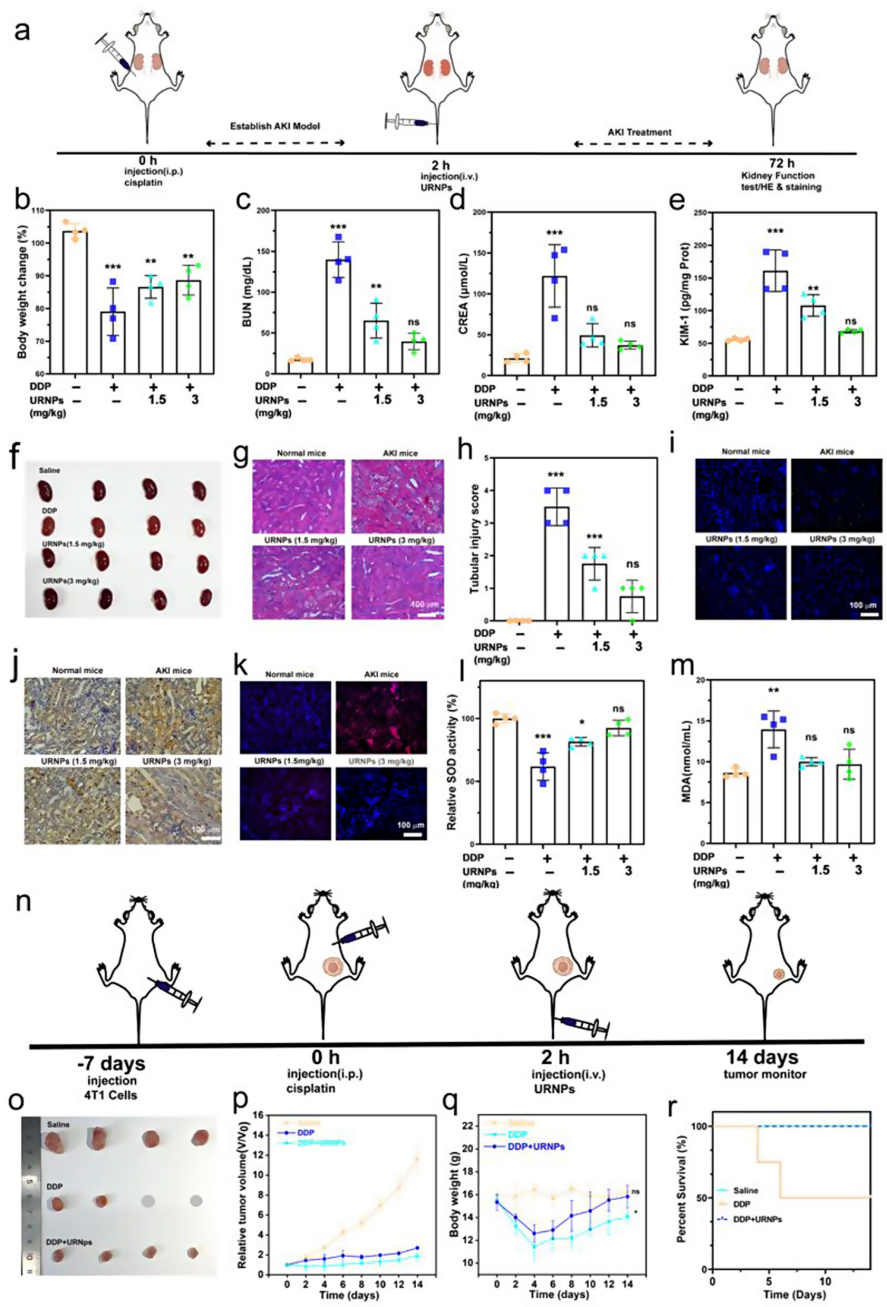
URNPs alleviate DDP-induced AKI in vivo. (**a**) Schematic illustration of AKI mice model establishment and treatment. The mice in four groups were treated with: saline, DDP (15 mg/kg), DDP (15 mg/kg) + URNPs (1.5 mg/kg), and DDP (15 mg/kg) + URNPs (3 mg/kg) (*n* = 4). (**b**) changes in body weight of mice with different treatments. P_(DDP)_ < 0.0001, P_(1.5 URNPs)_ = 0.0013, P_(3 URNPs)_ = 0.0037. (**c**) BUN. P_(DDP)_ < 0.0001, P_(1.5 URNPs)_ = 0.0059, P_(3 URNPs)_ = 0.2568; and (**d**) CREA levels in sera of mice after different treatments. P_(DDP)_ < 0.0001, P_(1.5 URNPs)_ = 0.0069, P_(3 URNPs)_ = 0.7805. (**e**) KIM-1 levels in renal homogenates. P_(DDP)_ < 0.0001, P_(1.5 URNPs)_ = 0.3034, P_(3 URNPs)_ = 0.7533. (**f**) Optical photographs of kidney. (**g**) Hematoxylin and eosin staining of kidney tissues from different group. Scale bar is 100 μm. (**h**) The tubular injury scores that were calculated according to the percentage of damaged tubules from H&E sections. P_(DDP)_ < 0.0001, P_(1.5 URNPs)_ = 0.0008, P_(3 URNPs)_ = 0.1469. (**i**) Fluorescence images of kidney tissues were collected from different groups stained by DAPI (blue fluorescence) and TUNEL (green fluorescence). Scale bar is 100 μm. (**j**) DAB staining of kidney tissues in different groups. The scale bar is 100 μm. (**k**) Fluorescence images of kidney tissues collected from different groups stained by DHE (red fluorescence) and DAPI (blue fluorescence). The scale bar is 100 μm. (**l**) SOD P_(DDP)_ < 0.0001, P_(1.5 URNPs)_ = 0.0107, P_(3 URNPs)_ = 0.4314. (**m**) MDA levels in different groups. P_(DDP)_ = 0.0015, P_(1.5 URNPs)_ = 0.5872, P_(3 URNPs)_ = 0.7514. (**n**) The tumor-bearing mice were divided into three groups: saline, DDP (6 mg/kg), DDP (6 mg/kg) + URNPs (3 mg/kg). Schematic illustration of tumor-bearing mice model establishment and therapy. (**o**)The optical images of tumors in different groups. (**p**) Relative tumor volume of mice in different groups. (**q**) Body weight changes of mice with different treatments. P_(DDP)_ = 0.018, P_(DDP + URNPs)_ = 0.6712. (**r**) The survival probability of mice in different groups. (The groups were analyzed using one-way ANOVA with multiple comparisons test. *n* = 4, mean ± SD, *p < 0.05, **p < 0.01, and ***p < 0.001)

Since URNPs have previously been proven to generate sufficient ROS in mimic TME under laser irradiation, we investigated whether URNPs could act as adjuvant to improve the efficacy of DDP. We chose mouse breast cancer (4T1) cells as a model due to the fact that DDP was commonly employed to treat breast cancer in clinic. CCK-8 assessment indicate that URNPs shows remarkable tumor cell suppression (Figure S13). By monitoring intracellular ROS levels, ROS were generated by URNPs, including ·OH. Due to the increased oxidative stress caused by URNPs in tumor cells, the mitochondrial membrane potential decreases. Damaged mitochondria activate cell apoptosis, potentiating tumor therapy therapeutic outcomes (Figure S14) [42]. Furthermore, ROS mediated by CDT or PDT can induce pyroptosis in tumor cells [43, 44]. It is speculated that ROS generated by URNPs could induce pyroptosis in tumor cells. Thus, the mechanism of adjuvant chemotherapy with URNPs is to synergistically induce apoptosis and pyroptosis by generating ROS. Based on the multiple programmed cell death induced by URNPs, which can be used to enhance the efficacy of DDP in tumor treatment. These results motivated us to further evaluate the effect of URNPs on the efficacy of DDP to tumor cells. It appears that introducing URNPs slightly increases the tumor-killing efficiency of DDP (Fig. 4a). We note that the tumor suppression efficiency could be further elevated by introducing near infrared (NIR) laser due to the generation of ^1^O_2_ by URNPs under laser irradiation (Figure S13). The cell viability of cells treated by DDP and 200 µM URNPs under laser irradiation decreased to 41.3%, which is significantly lower than cells treated by DDP alone (82.9%). However, no apparent effect of laser on the cell viability of tumor cells treated by DDP is observed. AM/PI co-staining analyses indicate that tumor cells treated by URNPs and DDP show stronger red fluorescence (dead cells) while weaker green fluorescence (live cells) compared to that treated by DDP alone, demonstrating the increased tumor killing efficiency caused by URNPs (Fig. 3b). We further analyzed the ROS level of 4T1 cells with different treatments. As expected, the introducing of URNPs increases the intensity of green fluorescence in DDP-treated cells, especially under laser irradiation (Fig. 3c, d). These results indicate that URNPs significantly increase the oxidative stress of tumor cells during DDP treatment. To demonstrate the simultaneous generation of ·OH and ^1^O_2_ by URNPs, we chose HKON-1r and SOSG as probes to monitor the ·OH and ^1^O_2_ levels. We observe that tumor cells treated by URNPs and DDP show significantly stronger fluorescence than cells treated by DDP in HKON-1r staining, suggesting the improved ·OH level caused by URNPs through Fenton-like reaction (Fig. 3e). Moreover, the intense green fluorescence could be only noted in cells treated by URNPs and DDP under laser irradiation in SOSG staining, confirming the successful generation of ^1^O_2_ by URNPs. Additionally, we detected mitochondrial membrane potential changes in 4T1 tumor cells by JC-1 to assess the mitochondrial dysfunction caused by increased oxidative stress. As expected, the strongest green fluorescence is observed in the DDP + URNPs + Laser group, suggesting that URNPs significantly reduces the mitochondrial membrane potential and induces numerous cells to undergo early apoptosis (Fig. 3f). These results demonstrate that URNPs successfully switch to ROS generation in TME and acts as promising DDP adjuvant for subsequent tumor therapy.

### In vivo biocompatibility and biodistribution of URNPs

To assess the biosafety of URNPs, we performed a hemolysis test. The hemolysis rate of URNPs (400 µM) was less than 5%, indicating high biosafety of URNPs (Figure S15). To further investigate in vivo toxicity, we intravenously injected URNPs (3 mg/kg) into healthy mice and collected blood samples to analyze the liver and renal function. The blood biochemical indexes, including aspartate aminotransferase (AST), alanine aminotransferase (ALT), blood urea nitrogen (BUN), and creatinine (CREA) are within the normal level. Meanwhile, the blood routine analyses show no significant difference between the URNPs-treated group and the saline-treated group (Figure S16). These results demonstrate the high biocompatibility of URNPs, making them ideal options for clinical DDP adjuvants that can be utilized for subsequent therapy. To confirm the URNPs were targeted to the organ site, we conducted ICP-MS analysis, to quantify the amount of Ru ions in different organs and tumor. The results showed that URNPs could rapidly reach kidneys and tumors within 1 h with accumulation rates of 4.38%ID/g and 1.05%ID/g(Figure S17). It appears that URNPs effectively accumulated in the tumor site via the enhanced permeability and retention (EPR) effect. Meanwhile, URNPs can effectively accumulate in kidney, owing to their small size. Besides, the accumulation amount is decreased with the time extension (Figure S18). Considering the benefits of tumor growth inhibition and balancing the therapeutic effect of URNPs on kidneys, we chose to treat the tumor with a laser 1 h after injection of URNPs.

### URNPs alleviate DDP-induced AKI in vivo

AKI mice models were established by intraperitoneal injection of DDP with the dosage of 15 mg/kg into healthy mice. We note significant weight loss in saline-treated AKI mice, while the AKI mice treated by URNPs show similar weight to healthy mice (Fig. 4b). Moreover, it appears that the AKI mice treated by saline show higher nitrogen (BUN) and creatinine (CREA) levels compared to healthy mice, indicating abnormal renal function in AKI mice (Fig. 4c and d). The BUN and CREA levels decrease to the normal level with the assistance of URNPs, demonstrating the recovery of renal function. Additionally, the expression of kidney injury molecule-1 (KIM-1) was analyzed. The saline treated AKI mice show significantly higher levels of KIM-1 compared to URNPs treated mice, which is similar to healthy mice (Fig. 4e). These results indicate the relief effect of URNPs on renal injury. Hematoxylin and eosin (H&E) staining of renal tissues from saline-treated AKI mice show severe renal injury, as evidenced by numerous casts, dilated, and necrotic tubules (Fig. 4g). However, URNPs treated AKI mice exhibit the typical structure of renal tubular. To quantify the degree of renal injury, we calculated the tubular injury score based on the H&E staining (Fig. 4h) [16]. It appears that the score of saline treated AKI mice is obviously higher than the healthy mice and URNPs treated AKI mice, demonstrating the alleviation effect of URNPs on AKI. These results are strongly supported by TUNEL fluorescence staining and immunohistochemical 3,3’-diaminobenzidine (DAB) staining analyses on renal tissues (Fig. 4i and j). Both TUNEL and DAB staining images indicate that the signal intensities in saline-treated AKI mice are significantly higher than that in URNPs-treated AKI mice. Specifically, the apoptosis ratios of renal cells are decreased with the increase of URNPs concentration. To understand the relationship between ROS scavenging of URNPs and its alleviation effect on AKI, we measure the level of ROS in the kidney tissue by staining the kidney tissue with dihydroethidium (DHE). The renal tissues of the URNPs treated AKI mice show dramatically lower ROS level compared to the saline-treated one, directly revealing ROS scavenging of URNPs in the renal tissue (Fig. 4k). Since superoxide dismutase (SOD) played an important role in the elimination of excess ROS, we further measured the levels of the SOD and malondialdehyde (MDA) in kidney tissue The SOD level of saline treated AKI mice is significantly lower than that of URNPs treated AKI mice, proving the restoration of SOD caused by URNPs (Fig. 4l). On the contrary, the introducing of URNPs lead to the increased MDA level caused by DDP return to the normal level (Fig. 4m).

It was found that an AKI mice model obtained by administering a single high dose of DDP was incompatible with tumor patients’ doses. Most cancer patients treated with cisplatin in the clinic receive low-dose cisplatin to minimize the risk of nephrotoxicity and maximize antitumor effectiveness. Patients receiving cisplatin for solid tumor treatment are typically given it chronically at a dose of less than 10 mg/kg [45]. Motivated by the AKI alleviation activity of URNPs, we verified whether URNPs could work as a DDP adjuvant to alleviate AKI during high-dosage DDP treatment. Thus, we chose a single intraperitoneal injection of 6 mg/kg of DDP (Fig. 4n). Specifically, 4T1 tumor-bearing BALB/c mice were randomly divided into three groups: Saline, DDP, and DDP + URNPs. Compared with the saline group, tumor growth is markedly inhibited in the DDP and DDP + URNPs group, owing to the high dosage DDP based anti-tumor efficacy. It should note that the tumor suppression effect of the DDP + URNPs group is slightly higher than that in the DDP group due to the mild CDT effect of URNPs (Fig. 4o, p, and Figure S19). Although high-dosage DDP effectively inhibits the growth of tumors, it results in an obvious decrease in body weight and even leads to the death of treated mice (Fig. 4q and r). Further, H&E staining of surviving mice revealed that DDP treated mice had renal tubular damage, including tubular dilation, loss of brush border, and epithelial degeneration (Figure S20). Excitingly, there are no apparent differences in the body weight and renal structure of the DDP + URNPs group compared to healthy mice. More importantly, all mice treated with high-dosage DDP using URNPs as adjuvant are alive during the treatment with high therapeutic efficacy, suggesting that URNPs work as efficient adjuvant to alleviate DDP-induced AKI with improved efficacy.

### URNPs improve efficacy of DDP in low dosage

**Fig. 5:**
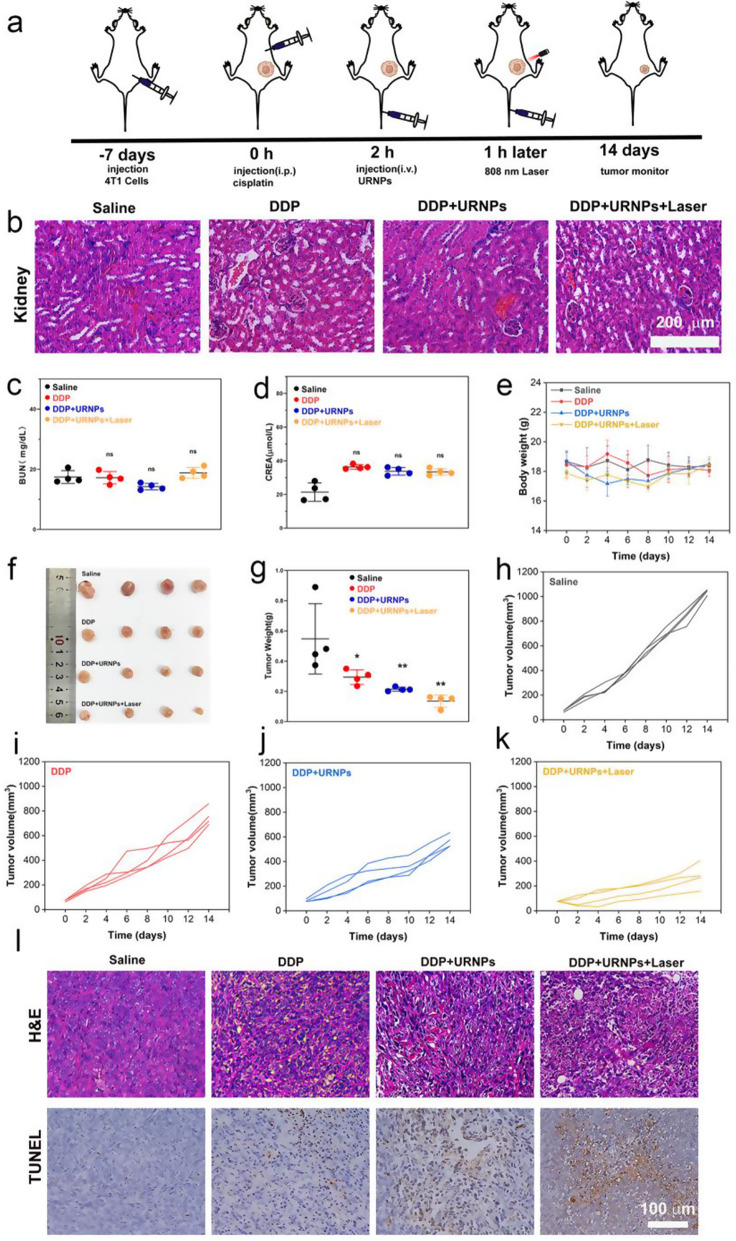
URNPs improve efficacy of DDP in safety dosage. (**a**) Schematic illustration of 4T1 tumor-bearing mice model establishment and therapy. The mice in four groups were treated with: saline, DDP (3 mg/kg), DDP (3 mg/kg) + URNPs (3 mg/kg), and DDP (3 mg/kg) + URNPs (3 mg/kg) + Laser (0.47 w/cm^2^) (*n* = 4). (**b**) H&E staining of kidney tissues from different groups. (**c**) BUN P_(DDP)_ = 0.9982, P_(DDP + URNPs)_ = 0.1127, P_(DDP + URNPs +Laser)_ = 0.7133. (**d**) CREA levels of mice after the treatments. P_(DDP)_ = 0.5978, P_(DDP + URNPs)_ = 0.7051, P_(DDP + URNPs +Laser)_ = 0.6578. (**e**) Body weight changes of mice with different treatments (**f**) Photograph of tumors after treatments on day 14 (*n* = 4). (**g**) Weight of tumors collected from tumor-bearing mice. P_(DDP)_ = 0.05, P_(DDP + URNPs)_ = 0.0098, P_(DDP + URNPs +Laser)_ = 0.002. Tumor growth curves of mice in the groups of (**h**) saline, (**i**) DDP, (**j**) DDP + URNPs, and (**k**) DDP + URNPs + Laser. (**l**) H&E and TUNEL staining of tumor tissues from different groups. Scale bar is 100 μm. (The groups were analyzed using one-way ANOVA with multiple comparisons test. *n* = 4, mean ± SD, *p < 0.05, and **p < 0.01)

Although DDP at 6 mg/kg appears to be an effective cancer treatment in vivo experiments with tumor-bearing mice, it also leads to inevitable renal toxicity and mortality. The goal of DDP therapy is to kill the cancer, thus prolonging the patient’s life. In order to maximize the translation of experimental progress into clinical use, we increased the frequency of DDP use and decreased the dose to mimic clinical administration. Therefore, DDP doses of 3 mg/kg once a week were chosen (Fig. 5a). Four groups of 4T1 tumor-bearing mice were selected: Saline, DDP, DDP + URNPs, and DDP + URNPs + Laser. We noted that all mice show typical renal tubular structures without any damage (Fig. 5b). Besides, the BUN and CREA levels in all groups are similar to control group, indicating the limited effect of low DDP dosage on renal function (Fig. 5c and d). It should note that no apparent changes in body weight during the 14-day treatment period (Fig. 5e). These results demonstrate that reduction of DDP dosage can effectively limit the occurrence of AKI. Unfortunately, the inhibition of DDP on tumor growth are significantly reduced. Compared to the mice treated by DDP, the tumor growth of mice treated by both DDP and URNPs is markedly inhibited (Fig. 5f-k and Figure S21). The inhibition rate in DDP + URNPs and DDP + URNPs + laser group increased from 27.76% (DDP group) to 52.67% and 74.77% (Figure S22). To further assess the therapeutic effects of various treatments, tumors from different groups are excised for H&E and TUNEL staining. Despite evident cell apoptosis/death in the DDP group, the highest tumor cell necrosis ratio occurred in the DDP + URNPs + Laser group (Fig. 5l). This phenomenon could be attributed to the enhanced oxidative stress induced by the URNPs-based ROS generation in tumors. In order to prove this speculation, we further treated tumor bearing mice by URNPs. The DHE staining of tumor tissues treated by URNPs indicate that URNPs could successfully mediate ROS generation in vivo, especially under laser irradiation. Meanwhile, TUNEL staining images reveal a large number of apoptotic cells, confirming that URNPs may improve DDP tumor efficacy via ROS production to induce cell apoptosis. The tumor growth of mice treated by URNPs are effectively inhibited due to the effective CDT and PDT of URNPs (Figure S23 and S24). Thus, it is a practical approach to assist DDP efficacy by inducing apoptosis through URNPs generating ROS in vivo. Furthermore, we do not observe any histopathological abnormalities in the major organs in all groups, indicating negligible adverse effects of URNPs on the mice (Figure S25 and S26). These results indicate the significant potential of URNPs with CDT/PDT synergy therapy activity to improve anti-tumor efficacy of DDP with high biocompatibility. Motivated by the low dose of 3 mg/kg of nontoxic DDP once a week, we chose to continue to increase the frequency of administration. To further evaluate whether URNPs could protect the kidneys from cisplatin nephrotoxicity, DDP doses of 3 mg/kg twice a week were chosen. We observe that URNPs treatment successfully improves the survival of mice treated by DDP with high dosage (Figure S27).

## Conclusion

In summary, we successfully synthesized URNPs that can be used as switchable ROS scavenger/generator respond to the tumor microenvironment. Both in vitro and in vivo experiments demonstrated that in a neutral physiological environment, excellent multiple enzymes ensure URNPs to protect renal cells and tissues by reducing DPP-induced ROS level. Once URNPs enter tumor tissue, it switch to ROS generator based on the H_2_O_2_ mediate oxidation. This unique feature endow URNPs to improve the oxidative stress of tumor during DDP treatment via ^1^O_2_ generation under laser irradiation and ∙OH through a Fenton-like reaction, which significantly improve DPP therapeutic effect in vitro and in vivo. Therefore, this TME-responsive ROS scavenger/generator, that work as adjuvant therapeutic agent to minimize side effects while enhance the efficacy of chemotherapy drugs, provides a new avenue to chemotherapy and facilitates clinical tumor therapy.

### Electronic supplementary material

Below is the link to the electronic supplementary material.

Supplementary Material 1

## Data Availability

Data is provided within the manuscript or supplementary information files.
