# Supplementary material for: Differentiated management of ROS level in tumor and kidney to alleviate Cis-platinum induced acute kidney injury with improved efficacy
Source: J Nanobiotechnology. 2024 Jul 24;22:436. doi: 10.1186/s12951-024-02710-2 (PMC11267679; doi:10.1186/s12951-024-02710-2)
Supplement: Supplementary file 1 — Supplementary Material 1 [file 12951_2024_2710_MOESM1_ESM.docx]

**Differentiated Management of ROS Level in Tumor and Kidney to Alleviate Cis-platinum Induced Acute Kidney Injury with Improved Efficacy**

Shiqi Zhu^1^*^#^*, Linlin Huo^1^*^#^*, Jie Zeng^1^, Rong Chen^2^, Yutong Sun^2^, Mingya Tan^1^, Mengke Fan^1^, Meiling Liu^1^, Jiayi Zhao^1^,Guoming Huang^2^, Yi Wang^1^, Zhibo Xiao^3*^, and Zhenghuan Zhao^1*^

^1^ College of Basic Medical Sciences, Chongqing Medical University, Chongqing 400016, China

^2^ College of Biological Science and Engineering, Fuzhou University, Fuzhou 350116, P. R. China

^3^ Department of Radiology, the First Affiliated Hospital of Chongqing Medical University, Chongqing 400016, China

*^#^*Shiqi Zhu and Linlin Huo contributed equally to this work.

*email: roddirck@cqmu.edu.cn and 202530@cqmu.edu.cn

**Supporting Information**

**Experimental Sections**

**Materials.** Ruthenium Acetylacetonate (Ru(acac)_3_), and 1,3-diphenylisobenzofuran (DPBF) were purchased from Adamas. Cisplatin (DDP) was purchased from Sigma-Aldrich. Benzyl ether (99%) and oleylamine (approximate C18-content 80-90%) were purchased from Acros. Oleic acid (tech. 90%) was purchased from Alfa Aesar. 1,2-distearoyl-sn-glycero-3-phosphoethanolamine-N-[amino (polyethylene glycol)-2000] (DSPE-PEG2000 amine) was purchased from J&K scientific. Tetrahydrofuran, n-hexane, ethanol, and chloroform were purchased from Sinopharm Chemical Reagent Co. Ltd. (Shanghai, China). 3,3',5,5'-Tetramethylbenzidine (TMB), methylene blue (MB), dulbecco's modified eagle medium F-12 (DMEM/F12) medium, roswell park memorial institute (RPMI) 1640 medium, fetal bovine serum (FBS), trypsin, penicillin−streptomycin, CCK-8 kit, calcein acetoxymethyl ester (calcein AM)/propidium iodide (PI) apoptosis detection agents, 2,7-dichlorodihydrofluoresceinn diacetate (DCFH-DA) , singlet oxygen sensor green (SOSG) reagent and 5,5,6,6-tetrachloro-1,1,3,3-tetraethyl-imidacarbocyanine iodide (JC-1) staining kit were purchased from Dalian Meilun Biotechnology Co. Ltd. HKON-1r was purchased from MedChemExpress. Kidney injury molecule-1 (KIM-1) ELISA kits were purchased from Quanzhou Jiubang Biotechnology Co., Ltd.

**Synthesis of ultrasmall ruthenium nanoparticles (URNPs).** URNPs were synthesized **via a thermo-decomposition method**. Briefly, Ru(acac)_3_ (112.80 mg, 0.28 mmol), 1,2-hexadecanediol (250.000 mg, 0.001 mmol), oleylamine (100 μL), and oleic acid (400 μL) were added to dibenzyl ether (10 mL). The mixture was evacuated from air and replenished with nitrogen. Then the mixture was heated at 300 °C for 1 hour. After cooling for half an hour, the product was collected and washed with ethanol and then dispersed in 6 mL of tetrahydrofuran.

**Surface functionalization of URNPs by DSPE-PEG.** To transfer URNPs into aqueous solution, the URNPs were modified with DSPE-PEG. URNPs (200 μL, 5mg/mL) and DSPE-PEG (40 μL, 50 mg/mL) in chloroform were mixed by sonication for 5 min. Then, the solution was evaporated slowly. The sample was re-dispersed in water by sonication after the chloroform was completely removed under a vacuum. The concentration of Ru ions in the solution was calculated by ICP-MS.

**The SOD-like activity of URNPs.** O_2_ ^·−^was produced by irradiating the solution of riboflavin and methionine. NBT, as a specific probe, was selected to detect the O_2_ ^·−^. Specifically, a mixture of methionine (130 mM), riboflavin (200 μM), nitrotetrazolium blue chloride (NBT) (750 μM), and different concentrations (0, 5, 10, 25, and 50 μg/mL) of URNPs was irradiated with a 365 nm UV lamp for 15 minutes in pH 7.4 PBS buffer (0.01 M). After irradiation, the absorbance was recorded at 560 nm by a microplate reader. Using the same method as above, DMPO was chosen as the spin trapping agent to evaluate the effect of different concentrations (0, 2.5, and 25 μg/mL) of URNPs in scavenging O_2_ ^·−^ the electron spin resonance (ESR) spectra.

**·OH Scavenging Ability of the URNPs.** ·OH was produced by a classical Fenton reaction. Salicylic acid (SA) was selected as a probe to evaluate the ·OH scavenging activity of URNPs. FeSO_4_ (1.8 mM) and H_2_O_2_ (5 mM) were mixed and stirred for 10 min. Different concentrations of URNPs (0, 2.5, 5, 25, and 50 μg/mL) and SA (1.8 mM) were added and stirred for another 5 min. Then, the absorbance of mixture was recorded at 510 nm by a microplate reader. By employing the same method, MB (100 μg/mL) was chosen as a probe to detect the ·OH level in a solution containing different concentrations of URNPs (0, 2.5, 5, 10, 25, and 50 μg/mL). The UV-Vis spectrophotometer was applied to measure the absorbance at 415 nm. Similarly, ·OH was captured by DMPO, which was used to evaluate the different concentrations (0, 2.5, and 25 μg/mL) URNPs of ·OH scavenging capacity by ESR spectrum.

**^1^O_2_ Scavenging Ability of the URNPs.** NaClO (7.5 mM) and H_2_O_2_ (2.5 mM) were mixed and stirred for 5 min. Then, different concentrations of URNPs (0, 50, and 100 μg/mL) and spin trapping agent TEMP were added and stirred for another 5 min. Afterward, ^1^O_2_ was detected using the ESR spectrum.

**The CAT-like activity of URNPs.** Catalase-like activity assays of URNPs under pH 7.4 conditions were carried out. The generated O_2_ was monitored by a dissolved oxygen meter (ST300D, OHAUS Corporation). Specifically, different concentrations of URNPs (0, 5, 10, and 20 μg/mL) and H_2_O_2_ (100 mM) were added to 10 mL of deionized water under stirring in a vacuum.

**^1^O_2_ Generation Ability of the URNPs.** 1,3-diphenylisobenzofuran (DPBF) was used to measure the generation of extracellular ^1^O_2_. Different concentrations of URNPs (5, 10, 15, 25, and 35 μg/mL), H_2_O_2_ (100 mM), and DPBF (10 mg/mL) were mixed in pH 6.5 PBS (0.01 M) and irradiated by 808nm laser (1 w/cm^2^) for 10min buffer. The absorbance at 432 nm before and after laser irradiation was measured by UV-vis spectrophotometer. Additionally, the mixture containing URNPs (25 μg/mL) and the trapping agent TEMP were irradiated to determine their effects on ^1^O_2_ production by ESR spectrum at presence or absence of H_2_O_2_ (100 mM).

**·OH Generation Ability of the URNPs.** 3,3',5,5' -tetramethylbenzidine (TMB) was chosen as a specific probe for the detection of ·OH. Different concentrations of URNPs (0, 2.5, 5, 7.5, 10, and 25 μg/mL), H_2_O_2_ (10 mM), and TMB (10 mg/mL) were mixed and stirred for 10 minutes in pH 6.5 PBS (0.01 M) solution. The absorbance at 652 nm was measured by UV-vis spectrophotometer. Additionally, URNPs (25 μg/ml), H_2_O_2_ (10 mM), and DMPO were mixed with or without laser irradiation (1 w/cm^2^) for 10 min to determine ·OH generation by URNPs.

**Cell culture.**Murine breast cancer 4T1 cells and Human Kidney Tubular Epithelial Cells (HK-2 cells) were obtained from the Chinese Academy of Sciences cell bank and cultured at 37 °C under 5% CO_2_. HK-2 cells were grown in a DMEM/F12 medium (10% FBS and 1% penicillin/streptomycin). 4T1 cells were grown in an RPMI-1640 medium (10% FBS and 1% penicillin/streptomycin).

**Cytotoxicity evaluation of HK-2 cells.** The HK-2 cells were cultured in a 96-well plate with a density of 1×10^4^ cells per well for 24 h. After washing the cells with PBS, different concentrations (0, 3.125, 6.25, 12.5, 25, 50, 100, and 200 μM) of URNPs were added and cultured for another 24 h. Finally, the CCK-8 kit was used to measure the cell viability. To verify the protective effect of URNPs in HK-2 cells, different concentrations (0, 3.125, 6.25, 12.5, and 25 μM) of URNPs and DDP (7.5 μM) were co-incubated with HK-2 cells. Similarly, different concentrations (0, 2.5, 5, 7.5, 10, 20, and 40 μM) of DDP and URNPs (100 μM) were incubated with HK-2 cells to test cytotoxicity. DDP alone group was a control group.

**Cytotoxicity evaluation of 4T1 cells.** Firstly, the 4T1 cells were cultured in a 96-well plate with a density of 1×10^4^ cells per well for 24 h. After washing the cells with PBS, the 4T1 cells were incubated with different concentrations (0, 6.25, 12.5, 25, 50, 100, 200, 300, and 400 μM) of URNPs for 20 h. These cells were treated with or without laser 808 nm irradiation (1 w/cm^2^) for 5 min. After another 4 h of incubation, the CCK-8 kit was used to measure cell viability. Additionally, we demonstrated that URNPs enhanced the efficacy of DDP in 4T1 cells. Briefly, 5 μM of DPP and different concentrations (0, 3.125, 6.25, 12.5, 25, 50, 100, and 200 μM) of URNPs were co-incubated in 4T1 cells, and these cells were irradiated by the 808 nm laser (1 w/cm^2^) for 5 min or none. The CCK-8 kit was used to measure cell viability.

**Calcein AM and PI Staining of HK-2 Cells.** Firstly, the 4T1 cells were cultured in a 96-well plate with a density of 1×10^4^ cells per well for 24 h. After washing the cells with PBS, 7.5 μM of DPP and different concentrations (0, 100, and 200 μM) of URNPs were co-incubated in HK-2 cells. The PBS-treated group was used as a control. The live and dead cells were stained with calcein-AM/PI for 30 min. Cells were washed with PBS three times to remove the unloaded probe, and images were observed using the fluorescence microscope (Leica DM 3000).

**Calcein AM and PI Staining of 4T1 Cells.** We divided into 5 groups: PBS group, Laser group, DPP group, DPP + URNPs group, and DPP + URNPs + Laser group. 4T1 Cells cells were incubated in confocal microscope dishes for 24 h, and the cell density was 1 × 10^4^ cells/ well. After washing the cells with PBS, the cells were incubated with PBS, laser, DPP, or DPP + URNPs (DDP: 5 μM, URNPs: 200 μM) for a further 20 h. Then, these cells were irradiated by an 808 nm laser (1 w/cm^2^) for 5 min or none. After another 4 h of incubation, the live and dead cells were stained with calcein-AM/PI for 30 min. Cells were washed with PBS three times to remove the unloaded probe, and images were observed using the fluorescence microscope (Leica DM 3000).

**Intracellular ROS Scavenging Detection (HK-2 Cells).** To assess the ROS Scavenging ability of URNPs, fluorescent probes (DCFH-DA) were used to detect intracellular ROS. In addition, Rosup was used to generate intracellular ROS. 4T1 Cells cells were incubated in confocal microscope dishes for 24 h, and the cell density was 1 × 10^5^ cells/ well. Then, DCFH-DA (5 μM) was added to cells treated with ROSUP (0.25 mg/mL) and different concentrations (0, 100, and 200 μM) of URNPs. After 40 min incubation, the cells were washed with PBS three times, and finally imaging was conducted by using the fluorescence microscope (Leica DM 3000).

Furthermore, intracellular oxidative stress was also induced by DDP. HK-2 cells were incubated with 7.5 μM of DDP and different concentrations (0, 100, and 200 μM) of URNPs for 7 h. Subsequently, DCFH-DA (5 μM) was added and cultured for 40 min. Cells were washed with PBS three times to remove the unloaded probe. Imaging was performed with a fluorescence microscope (Leica DM 3000) and subjected to flow cytometry analysis to quantify intracellular ROS levels.

**Intracellular ROS generation Detection (4T1 Cells).** To evaluate the ability of URNPS to enhance DDP ROS production by 4T1 cells, we divided into 5 groups: PBS group, Laser group, DPP group, DPP + URNPs group, and DPP + URNPs + Laser group. 4T1 Cells cells were incubated in confocal microscope dishes for 24 h, and the cell density was 1 × 10^5^ cells/ well. After washing the cells with PBS, the cells were incubated with PBS, Laser, DPP, or DPP +URNPs (DDP: 5 μM, URNPs: 200 μM) for a further 7 h. Then, these cells were irradiated by an 808 nm laser (1 w/cm^2^) for 5 min or none. The cells were cultured with DCFH-DA (5 μM) for a further 40 min. The cells were washed with PBS three times, and finally, imaging was conducted using the fluorescence microscope (Leica DM 3000).

To further distinguish the type of ROS, we utilized HKON-1r (10 mM) and SOSG (5 mM) as fluorescence probes to monitor the intracellular ·OH and ^1^O_2_. Similar to the above steps, imaging was conducted using the fluorescence microscope (Leica DM 3000).

**Mitochondrial Membrane Potential (MMP) Assay (HK-2 cells).** Mitochondrial membrane potential perturbation was measured with a JC-1 mitochondrial membrane potential assay kit. Firstly, the HK-2cells were cultured in a 96-well plate with a density of 1×10^4^ cells per well for 24 h. After washing the cells with PBS, 7.5 μM of DPP and different concentrations (0, 100, and 200 μM) of URNPs were co-incubated in HK-2 cells for 7 h. The HK-2 cells were stained with JC-1 working fluid for 30 min. The cells were washed with PBS three times, and finally, imaging was conducted using the fluorescence microscope (Leica DM 3000).

**Mitochondrial Membrane Potential (MMP) Assay (4T1 cells).** We divided into 5 groups: PBS group, Laser group, DPP group, DPP +URNPs group, and DPP + URNPs + Laser group. 4T1 Cells cells were incubated in confocal microscope dishes for 24 h, and the cell density was 1 × 10^5^ cells/ well. After incubation, the cells were incubated with PBS, Laser, DPP, or DPP +URNPs (DDP: 5 μM, URNPs: 200 μM) for a further 7 h. After washing the cells with PBS, these cells were irradiated by an 808 nm laser (1 w/cm^2^) for 5 min or none. The cells were cultured with JC-1 working fluid for a further 40 min. The cells were washed with PBS three times, and finally, imaging was conducted using the fluorescence microscope (Leica DM 3000).

**Hemolysis assay.** Fresh whole blood samples were obtained from BALB/C mice's orbital veins. To prepare pure erythrocytes, the blood was centrifuged at 3000 rpm and washed five times in PBS solution. Then, erythrocytes were mixed with deionized water, PBS, and different concentrations (0, 60, 80,100, 200, 300, and 400 μM) of URNPs, respectively. A mixed solution was incubated at 37 °C for 4 hours. Hemolysis was observed and calculated after centrifuging for 10 minutes at 10000 rpm. The hemolysis ratio was quantified by measuring the absorbance at 540 nm with a microplate reader.

**In vivo biocompatibility evaluation of URNPs.** URNPs were administered intravenously to healthy BALB/C mice at a dosage of 3 mg/kg. The mice injected with saline served as the control group. At 24 h after injection, all mice were sacrificed and then collected whole blood samples for hematology analysis.

**In Vivo Biodistribution.** BALB/C mice bearing tumors were intravenously injected with URNPs (3 mg/kg). The mice were sacrificed 1 h after injection and the major organs (heart, liver, spleen, lung, and kidney) and tumors were harvested for quantifying the percentage of URNPs in each organ. To further assess the accumulation of URNPs in kidney and tumor at different times, the mice were sacrificed at different times (0.5h, 1h, 2h, 24h) after injection, kidneys and tumors were harvested. Afterward, the samples were dissolved in aqua regia, and the concentration of Ru was measured by ICP-MS.

**AKI model establishment and treatment.** Animal experiments were conducted according to the protocol approved by the Institutional Animal Care and Use Committee of Chongqing Medical University (No. 2020320). All female BALB/c mice (6 -8 weeks old) were obtained from Chongqing Medical University Laboratory Animal Center. (1) To establish a model of DDP-induced acute kidney injury, BALB/C mice were given a single intraperitoneal injection of 15 mg/kg of DDP. Two hours after the model induction, DDP-induced AKI mice were intravenously injected with different dosages of URNPs (0, 1.5, and 3 mg/kg) (*n* = 4). Healthy control mice were injected intraperitoneally with an equal volume of saline. (2) To further assess the effect of URNPs on the survival rate of the low-dose DDP, BALB/C mice were divided into 4 groups: Saline group, URNPs group, DDP group, and DDP +URNPs group (*n* = 8). DDP was intraperitoneally injected at the dosage of 3 mg/kg (twice a week), and URNPs were intravenously injected at the dosage of 3 mg/kg (twice a week). Healthy control mice were injected intraperitoneally with an equal volume of saline. The survival curves of mice within 7 days under the dosage were drawn.

**In vivo therapeutic outcome of URNPs on AKI mice.** All mice were sacrificed to collect blood and kidney tissue. Renal function analyses and histological analyses were performed to assess URNPs therapeutic effect on AKI mice.

To be more specific, the blood samples were collected and centrifuged at 8000 rpm for 10 min at 4 °C. Analysis of blood creatinine (CREA) levels and blood urea nitrogen (BUN) levels was carried out.

To further detect renal biomarkers, kidneys were harvested and homogenized. Malondialdehyde (MDA) and superoxide dismutase (SOD) levels in the kidney were measured using an MDA and SOD assay kit, respectively. Moreover, kidney injury molecule-1 (KIM-1) ELISA kits were used to monitor the expression levels of kidney injury biomarkers.

Renal tissues were sectioned and stained with H&E in order to observe renal pathological changes and the extent of tubular damage. Renal tubular injury score: randomly choosing five fields of view and scoring semi-quantitatively according to the percentage of damaged tubules (0, no damage; 1, <25 % damage; 2, 25–50 % damage; 3, 50–75 % damage; 4, >75 % damage). DHE staining was performed on renal tissues to detect ROS levels.

**Terminal Deoxynucleotidyl Transferase-mediated dUTP-biotin Nick End Labeling (TUNEL) staining.** Apoptotic cells were detected by Diaminobenzidine (DAB) biotin-labeled or fluorescein-labeled TUNEL kits after tissue sectioning. The nuclei of positive apoptotic cells labeled with DAB were brownish-yellow, and fluorescein-labeled apoptotic cells were green. The images were conducted using the fluorescence microscope (Leica DM 3000).

**In Vivo Tumors Therapy.** The 4T1 tumors were induced by subcutaneous injection of (2.5 × 10^6^) cells onto the back of each Female BALB/c mice (6 - 8 weeks old). 4T1 tumor-bearing BALB/c mice were evaluated for the effect of URNPs on tumors when the tumors grew to an average volume of 75-105 mm^3^. BALB/c mice were randomly divided into three groups (*n* = 4): healthy mice group, Laser group, and URNPs + Laser group. URNPs were intravenously injected at the dosage of 3 mg/kg (once a week). One-hour post-injection, the tumor was irradiated with a laser (0.47 w/cm^2^, 10 min). We recorded the body weight of mice and the tumor volume every other day. The tumor volumes were measured as length × (width)^2^ × 1/2. These mice were executed on day 14 to collect organs and tumors for histological analysis.

In order to further assess the effect of PDT treatment of URNPs on the efficacy of DDP therapy, the same experiment setup was applied to 4T1 tumor-bearing BALB/c mice. They were randomly divided into four groups (*n* = 4): Saline group, only DDP group, DDP + URNPs group, and DDP + URNPs + Laser group. DDP was intraperitoneally injected at the dosage of 3 mg/kg (once a week), and URNPs were intravenously injected at the dosage of 3 mg/kg (once a week). One-hour post-injection, the tumor was irradiated with a laser (0.47 w/cm^2^, 10 min). The body weight of mice and the tumor volume were recorded every other day.

To evaluate the effect of URNPs on the efficacy of DDP treated tumors and kidney protection, the same experiment setup was applied to 4T1 tumor-bearing BALB/c mice. BALB/c mice were randomly divided into three groups (*n* = 4): healthy mice group, DDP group, and DDP + URNPs group. DDP was single intraperitoneally injected at the dosage of 6 mg/kg, URNPs were intravenously injected at the dosage of 3 mg/kg. We recorded the body weight of mice and the tumor volume every other day. The tumor volumes were measured as length × (width)^2^ × 1/2. Furthermore, the survival curves of mice within 2 weeks under the dosage were drawn. These mice were executed on day 14 to collect organs and tumors for histological analysis.

**Statistical analysis.** Data were presented as the mean ± SD from the experiments. The two groups were analyzed using the Student's t-test. To analyze the difference between three or more groups, a one-way analysis of variance multiple comparisons was applied (GraphPad Prism 8.0, GraphPad Software, CA, USA). There was statistical significance between multiple groups as follows: * p < 0.05, **p < 0.01, and ***p < 0.001.





**Figure S1.** The TEM statistical chart of particle size distribution.


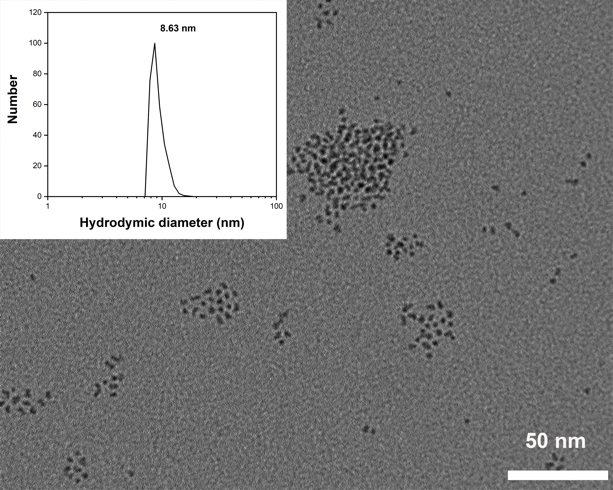


**Figure S2.** TEM image of URNPs after DSPE-PEG surface functionalization in aqueous. Scale bar is 50 nm. Insert hydrodynamic diameter analyses of URNPs.





**Figure S3.** Zeta potentials of URNPs.





**Figure S4.** Dynamic light scattering analyses of URNPs in ddH_2_O, PBS, and complete medium for 0, 24, and 72 hours.





**Figure S5.** UV-Vis spectra of ·OH scavenging capacity at different concentrations of URNPs by using MB as a probe.





**Figure S6.** UV-Vis spectra of ^1^O_2_ generation at different treatments of URNPs by using DPBF as probe.





**Figure S7.** CAT-like activity of URNPs. UV-Vis spectra of O_2_ generation levels with H_2_O_2_ and different concentrations of URNPs.





**Figure S8.** ESR spectra analyses of oxygen vacancy generation with URNPs after H_2_O_2_ treatment.


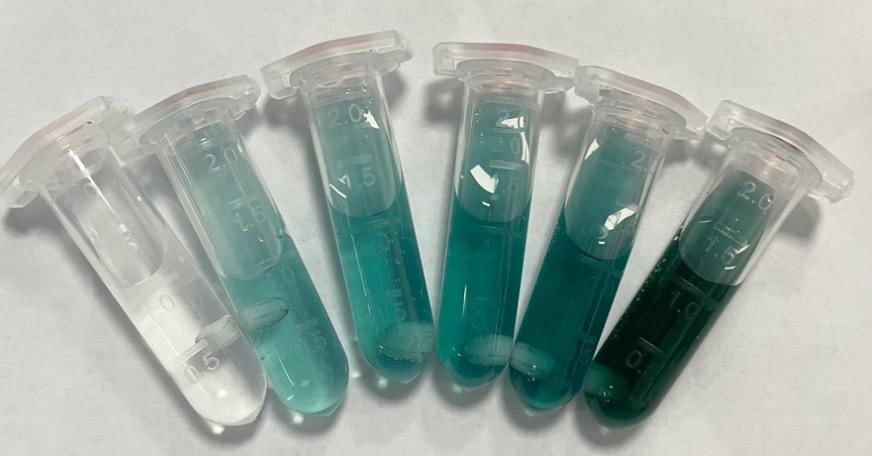


**Figure S9.** Optical photographs of ·OH generation at different concentrations of URNPs (0, 2.5, 5, 7.5, 10, and 25 μg/mL) by using TMB as a probe.


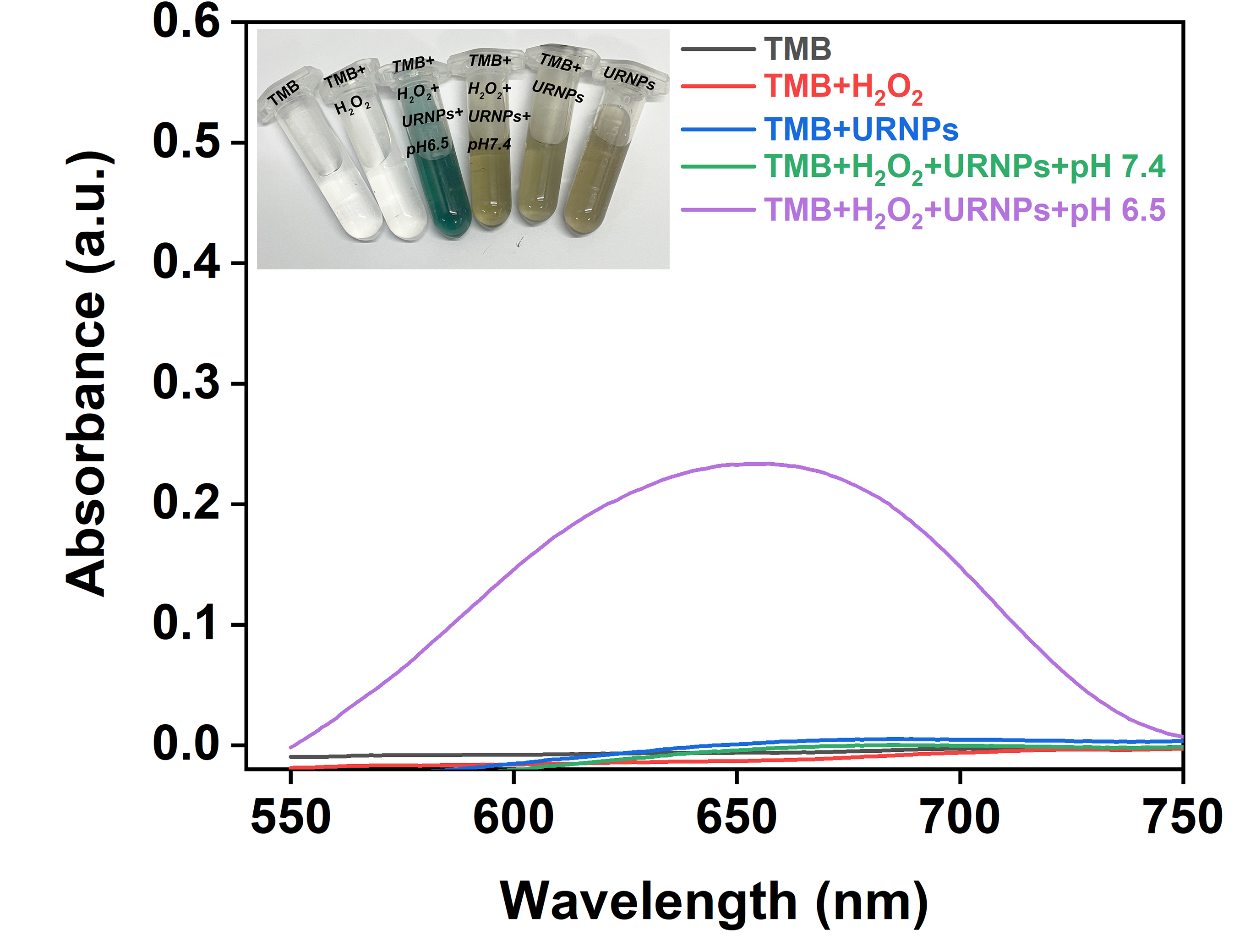


**Figure S10.** UV-Vis spectra of ·OH generation at different treatments of URNPs (25 μg/mL) by using TMB as a probe. Insert optical photographs of ·OH generation by using TMB as a probe.


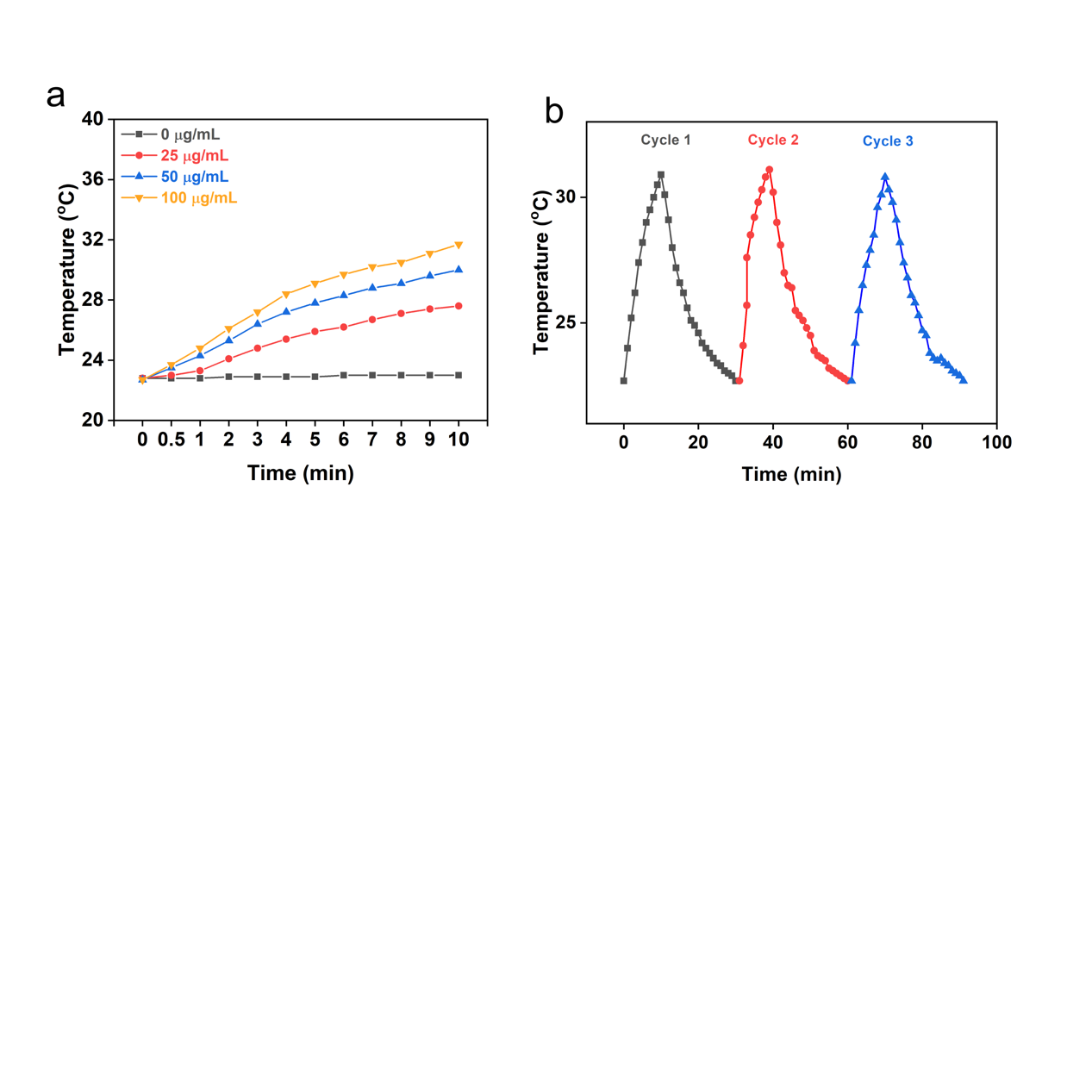


**Figure S11.** Photothermal properties of URNPs. (a) The temperature rise changes of different concentrations of URNPs (0, 25, 50, and 100 μg/mL) under 808 nm laser irradiation (0.47 w/cm^2^) for 10 min. (b) Irradiation cycles three times of URNPs (50 μg/mL) under 808 nm laser irradiation (0.47 w/cm^2^) It notes that URNPs show the same behavior in absorption spectra and heating curves before and after three irradiation cycles, showing good photothermal stability.


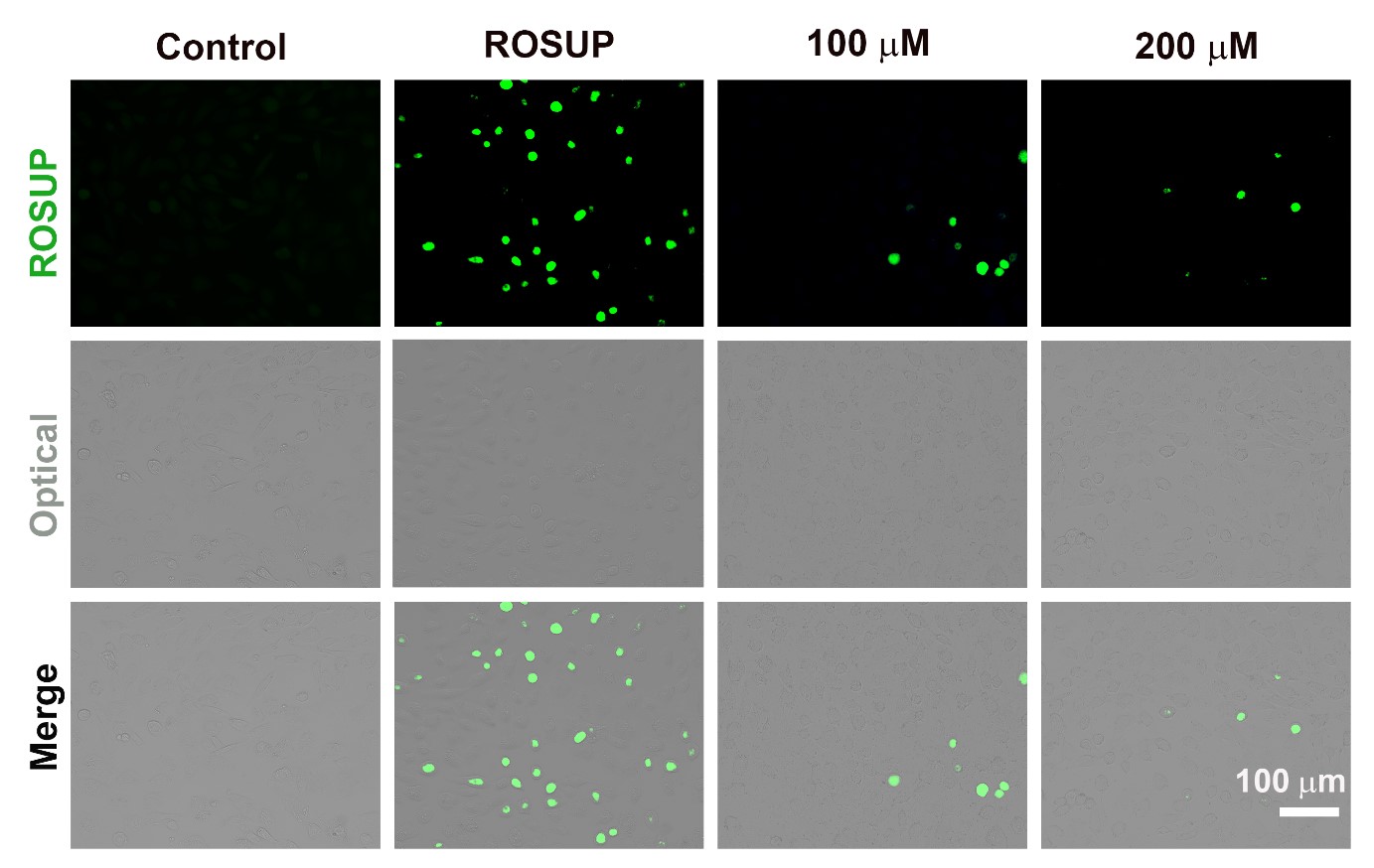


**Figure S12.** ROS Scavenging Activities of URNPs intracellular. Fluorescence images of HK-2 cells after treatment with ROSUP (0.25 mg/mL) and different concentrations (0, 100, and 200 μM) of URNPs to monitor ROS by using DCFH-DA as an indicator. Scale bar is 100 μm.





**Figure S13.** The cell viability of 4T1 cells treated by different concentrations of URNPs and with or without laser irradiation.

**
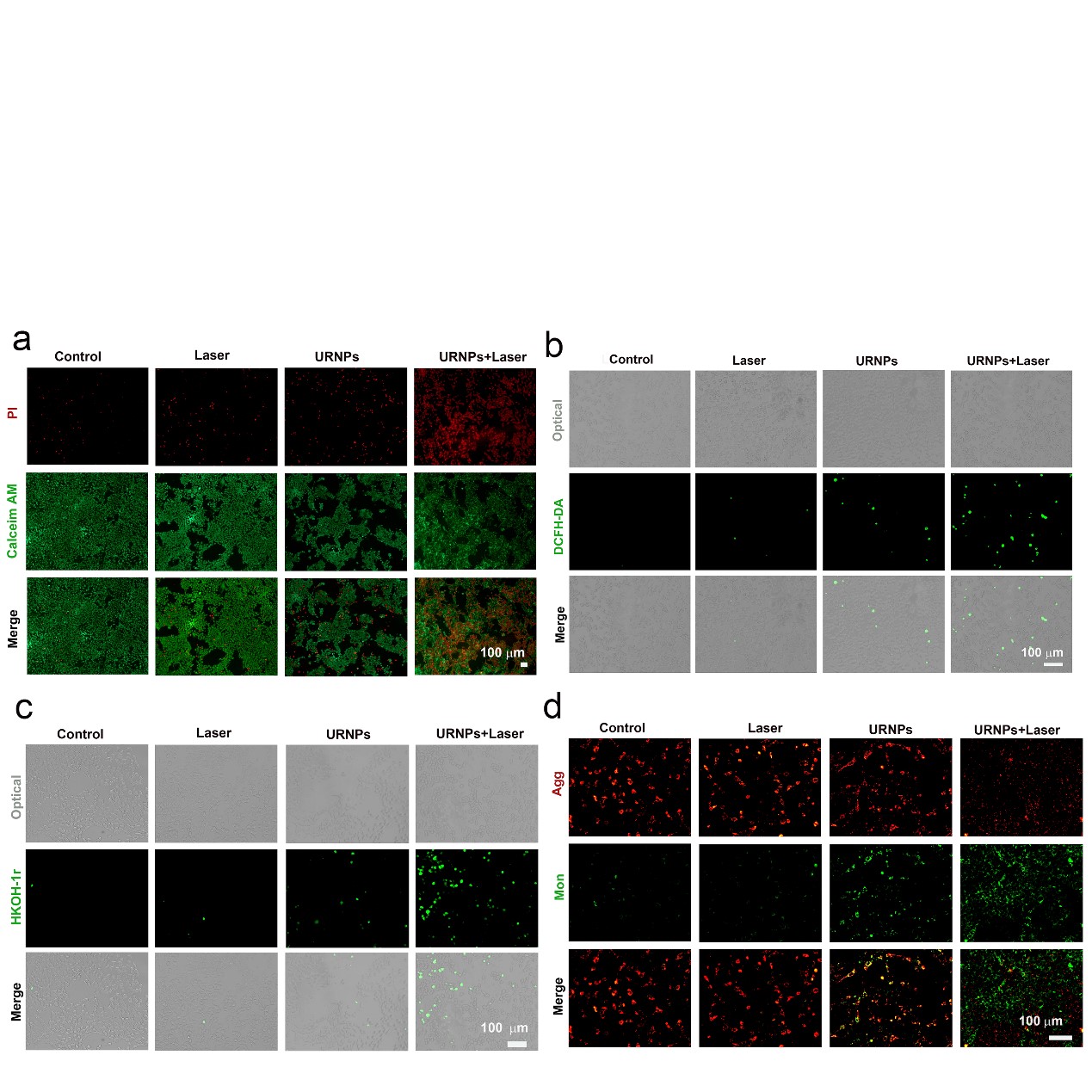
**

**Figure S14.** CDT and PDT effect of URNPs in 4T1 cells. (a) Live/dead cell staining tests in four groups: control, Laser (1 w/cm^2^), URNPs (200 μM), Laser (1 w/cm^2^) + URNPs (200 μM). Red, dead cells; green, live cells. Scale bar is 100 μm. (b) Fluorescence images of cells under different treatments to monitor ROS by using DCFH-DA as an indicator. Scale bar is 100 μm. (c) The intracellular ·OH was detected by HKON-1r as a fluorescence probe under different treatments. Scale bar is 100 μm. (d) Fluorescence images of cells under different treatments to monitor mitochondrial membrane potential measurement by JC-1 probes. Scale bar is 100 μm.


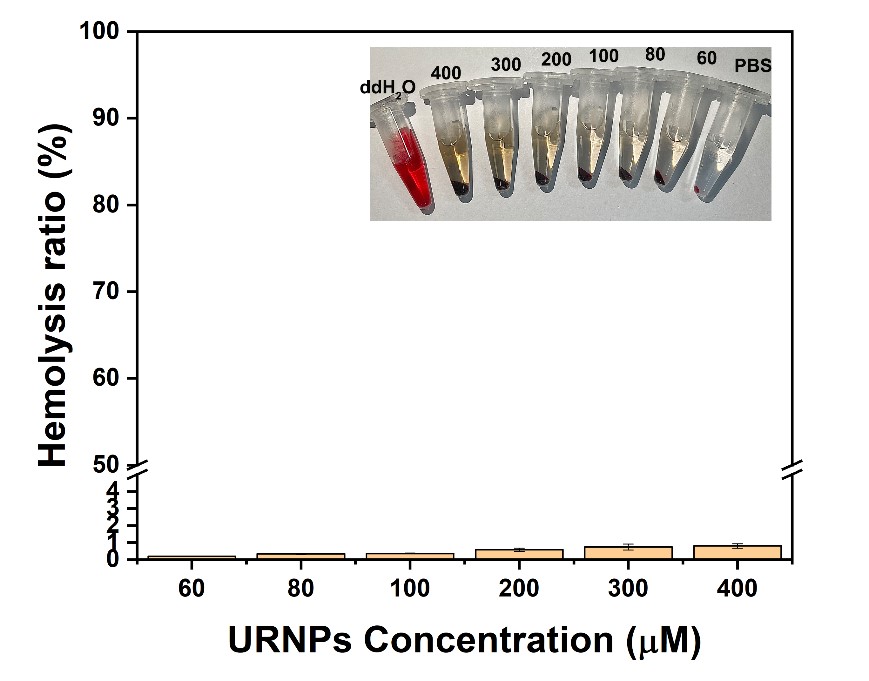


**Figure S15.** Hemolysis assay of different concentrations of URNPs. Deionized water is used as a positive control, and PBS as a negative control.


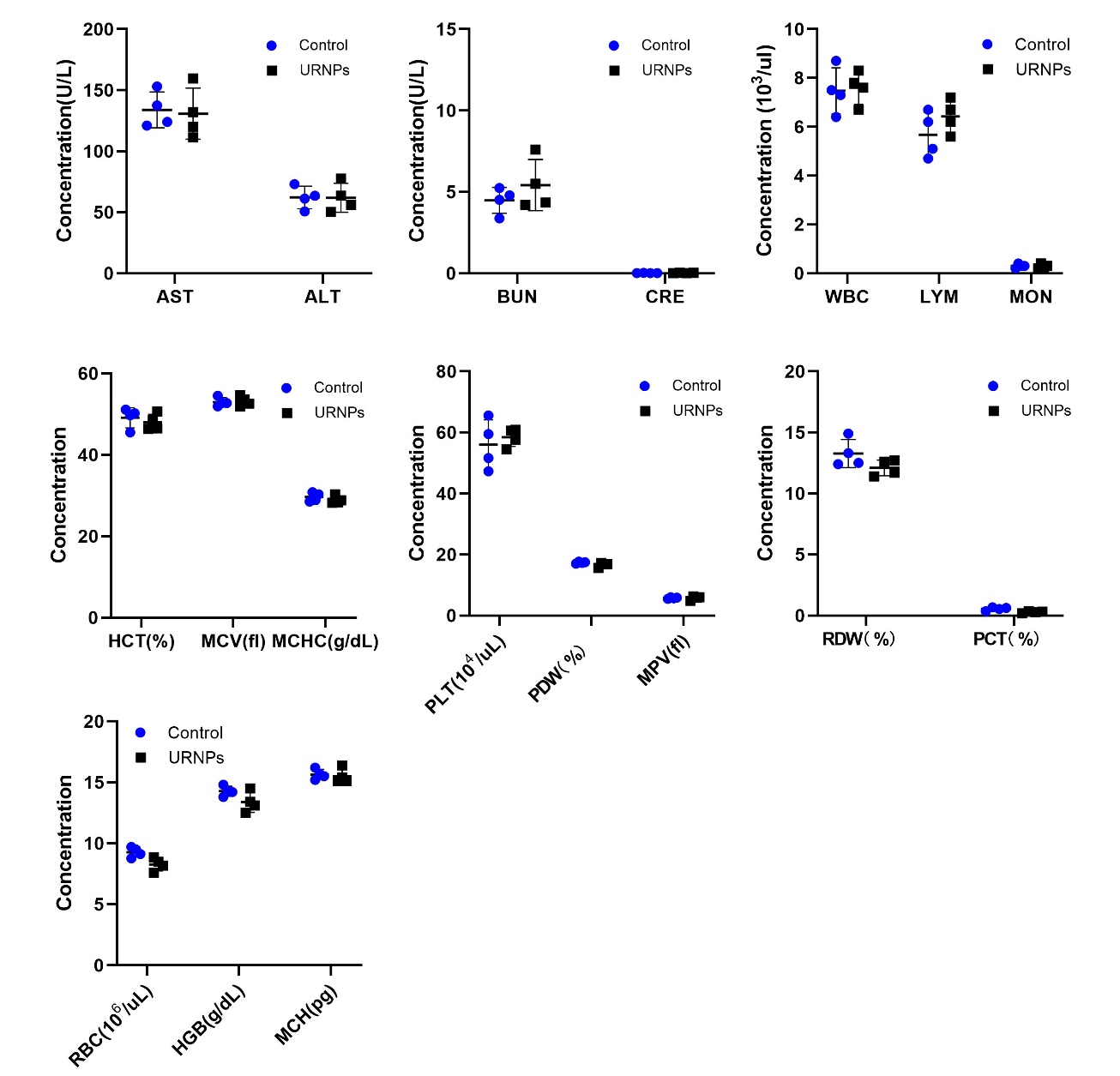


**Figure S16.** Quantitative analysis of blood biochemistry indices of mice treated with saline or URNPs (3 mg/kg) (*n* = 4).

*
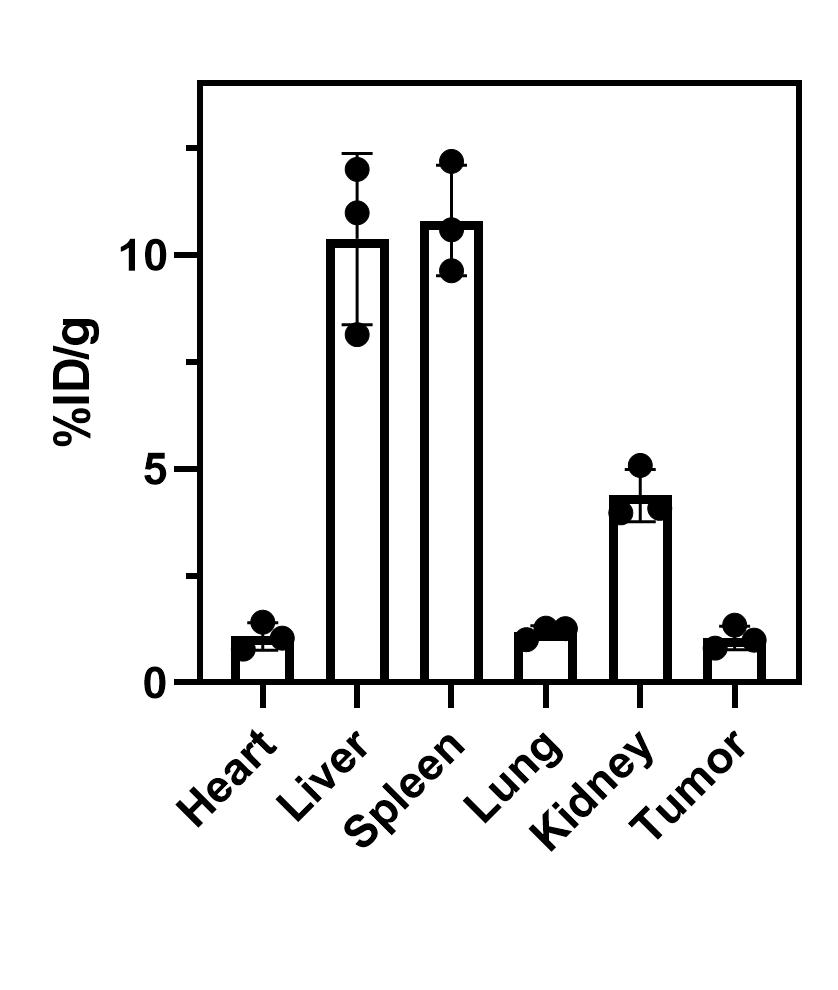
*

**Figure S17.** Biodistribution of Ru ions in tumor-bearing mice 1 h after intravenous injection of URNPs. (*n* = 3)


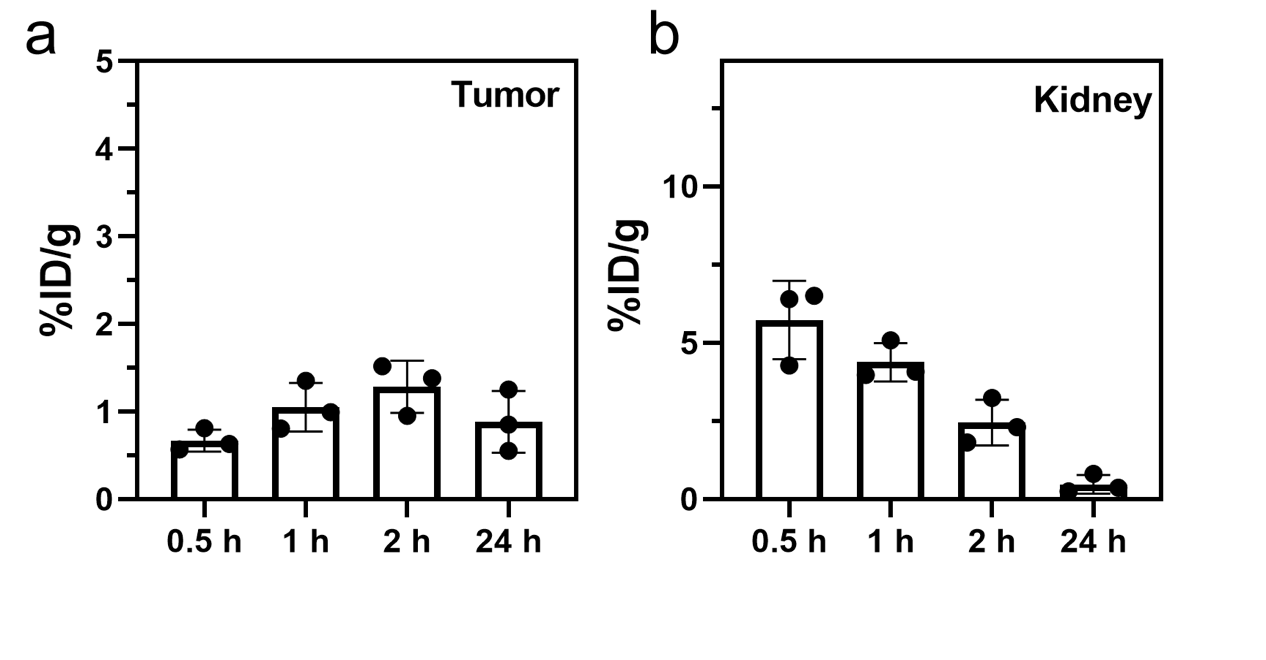


**Figure S18.** Accumulation of URNPs in tumor-bearing mice in (a) tumor and (b) kidney at various times after intravenous injection (*n* = 3).


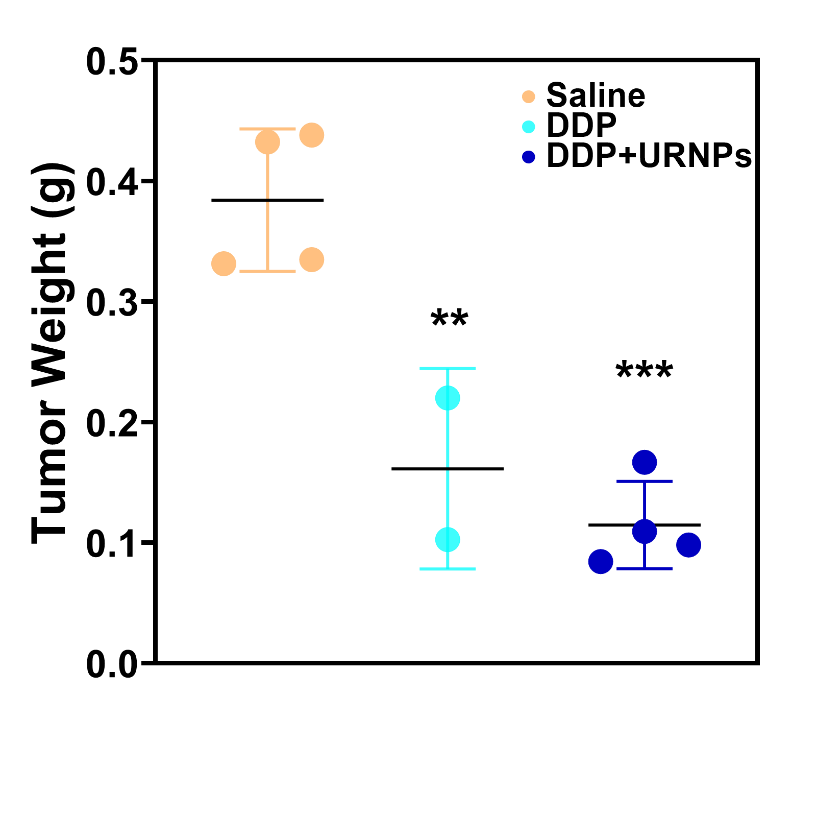


**Figure S19.** Weight of tumors collected from tumor-bearing mice in different treatments. P_(DDP)_ = 0.0057, P _(DDP + URNPs)_ = 0.0006, (One-way ANOVA with multiple comparisons test. *n* = 4, mean ± SD, **p* < 0.05, ***p* < 0.01, and ****p* < 0.001).


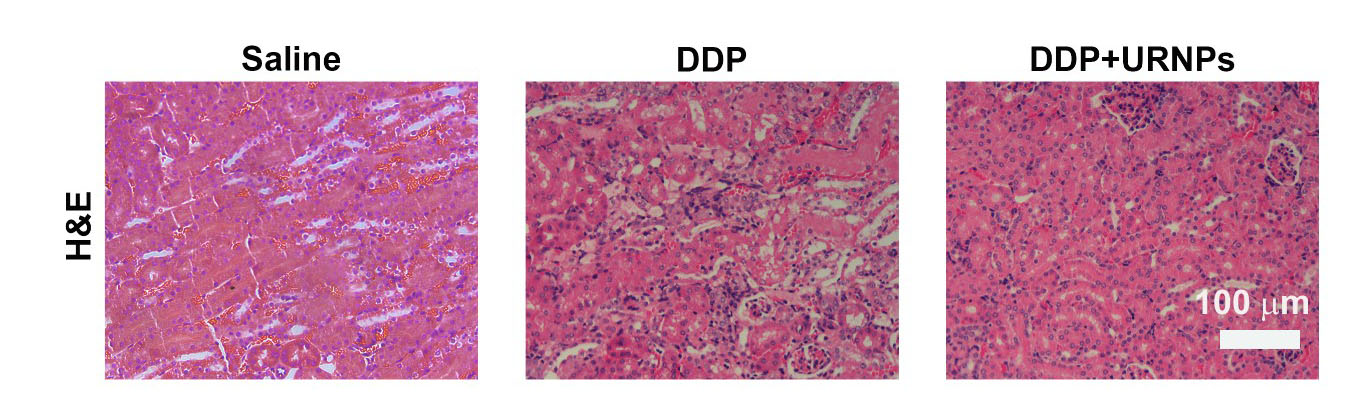


**Figure S20.** H&E staining of kidney tissues from different groups. Scale bar is 100 μm.





**Figure S21.** Relative tumor volume of mice in different groups. P_(DDP)_ = 0.0041, P _(DDP + URNPs)_ < 0.0001, P _(DDP + URNPs + Laser)_ < 0.0001 (One-way ANOVA with multiple comparisons test. (One-way ANOVA with multiple comparisons test. *n* = 4, mean ± SD, **p* < 0.05, ***p* < 0.01, and ****p* < 0.001).


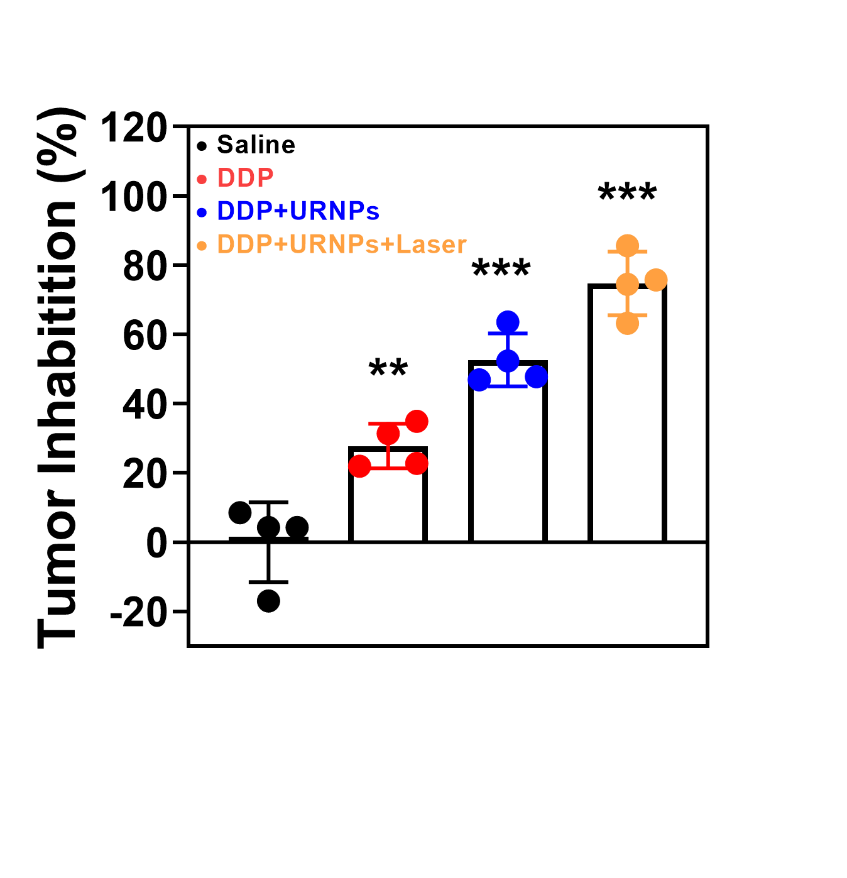


**Figure S22.** Tumor inhabitation rate of mice in different groups. P_(DDP)_ = 0.0041, P _(DDP + URNPs)_ < 0.0001, P _(DDP + URNPs + Laser)_ < 0.0001 (One-way ANOVA with multiple comparisons test. (One-way ANOVA with multiple comparisons test. *n* = 4, mean ± SD, **p* < 0.05, ***p* < 0.01, and ****p* < 0.001).


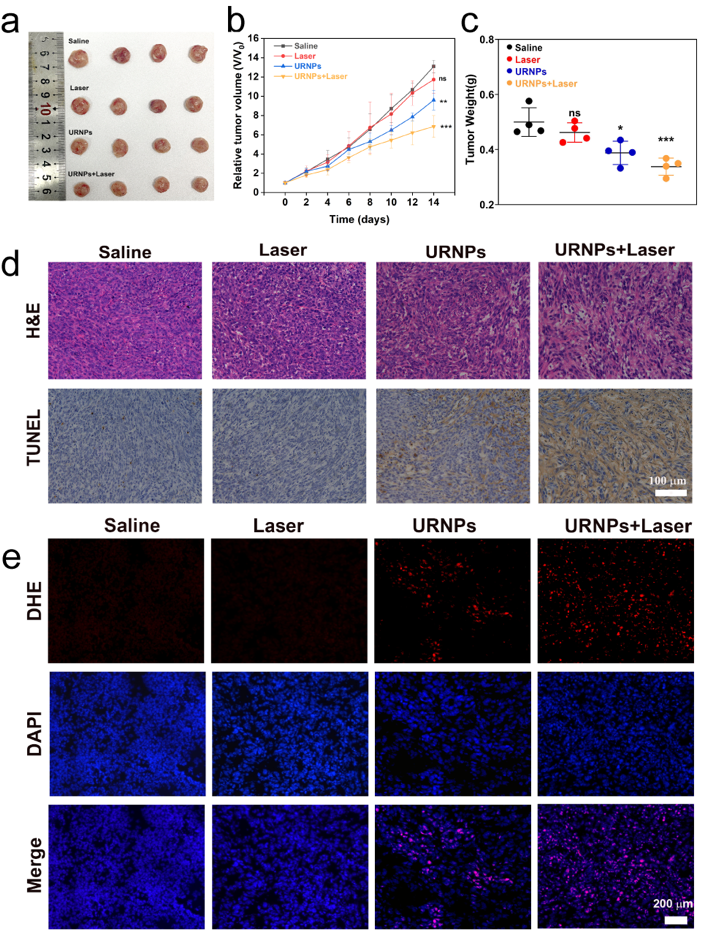


**Figure S23.** In *vivo* CDT and PDT of URNPs. The tumor-bearing mice in four groups were treated with saline, Laser (0.47 w/cm^2^), URNPs (3 mg/kg), Laser (0.47 w/cm^2^) + URNPs (3 mg/kg). (a) The optical images of tumors. (b) Relative tumor volume of mice in different groups. P_(Laser)_ = 0.3404, P_(URNPs)_ = 0.0034, P_(URNPs + Laser)_ = 0.0105. (c) Weight of tumors collected from tumor-bearing mice. P_(Laser)_ = 0.5789, P_(URNPs)_ = 0.0382, P_(URNPs + Laser)_ < 0.0001. (d) H&E and TUNEL staining of tumor tissues from different groups. Scale bar is 100 μm. (e) DHE staining of tumor tissues from different groups. Scale bar is 200 μm. (One-way ANOVA with multiple comparisons test. *n* = 4, mean ± SD, **p* < 0.05, ***p* < 0.01, and ****p* < 0.001).


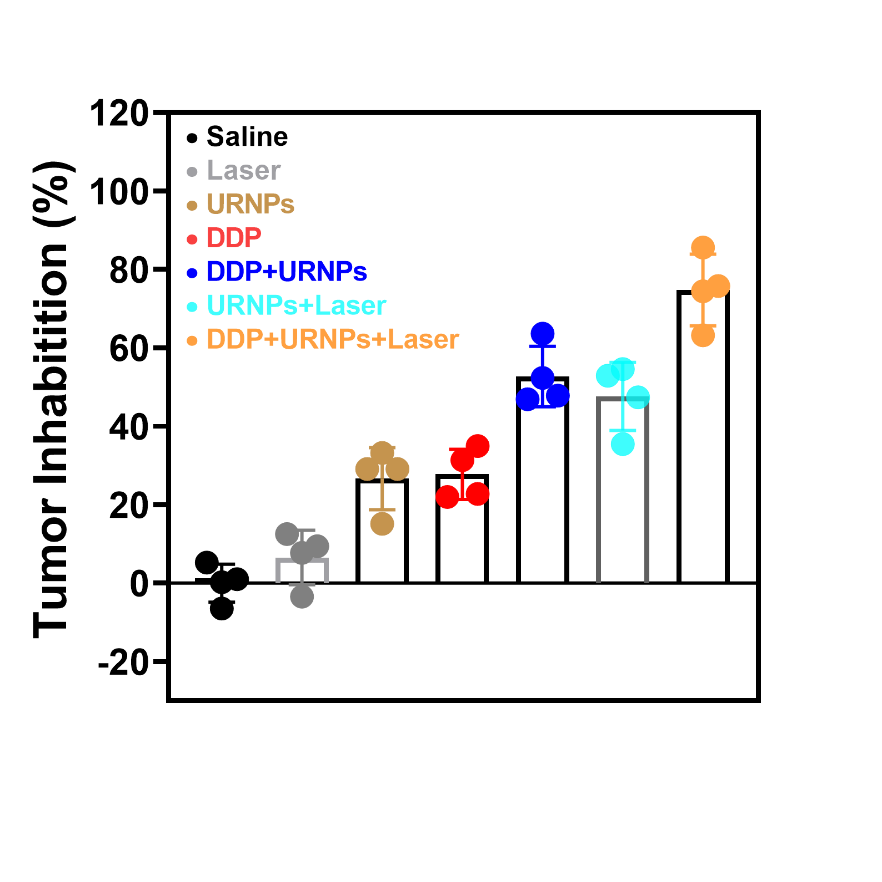


**Figure24.** Tumor inhabitation rate of mice in different groups.


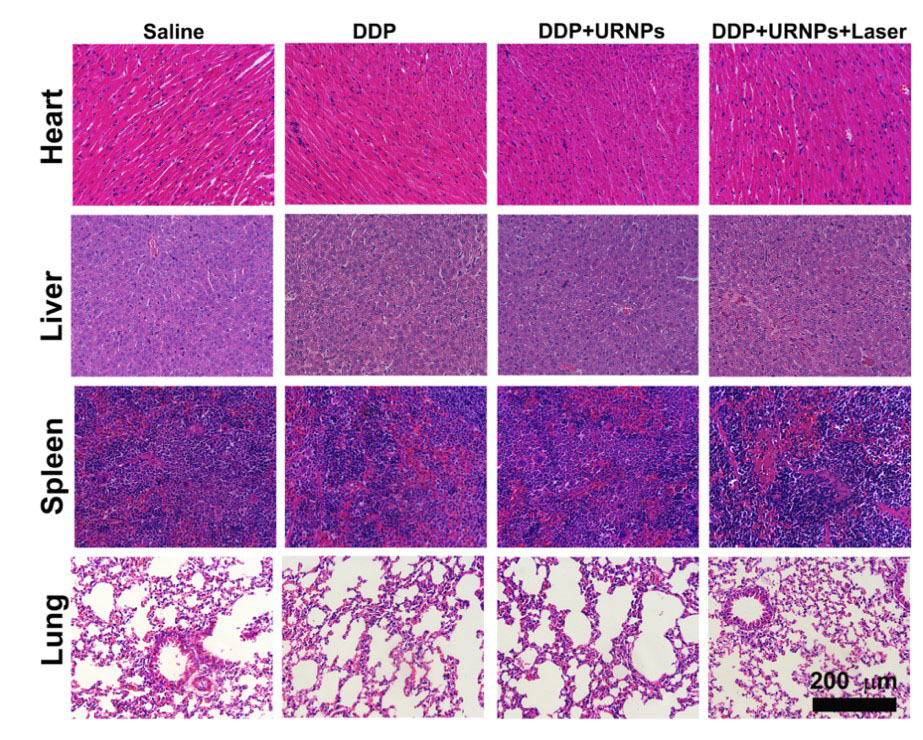


**Figure S25.** Collection of organs after a 14-day cycle of different treatments in tumor-bearing mice. Hematoxylin and eosin staining of primary organs (the heart, liver, spleen, and lung) showed no sign of toxicity. Scale bar is 200 μm.

**
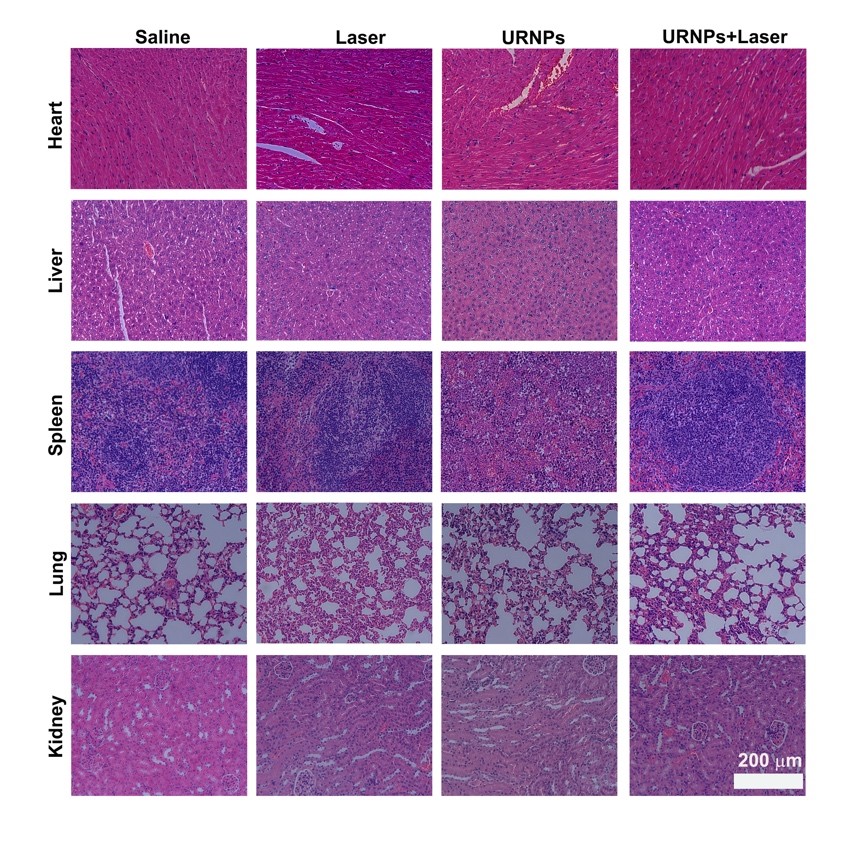
**

**Figure S26.** Collection of organs after a 14-day cycle of different treatments in tumor-bearing mice. H&E staining of primary organs (the heart, liver, spleen, lung, and kidneys) showed no sign of toxicity. Scale bar is 200 μm.


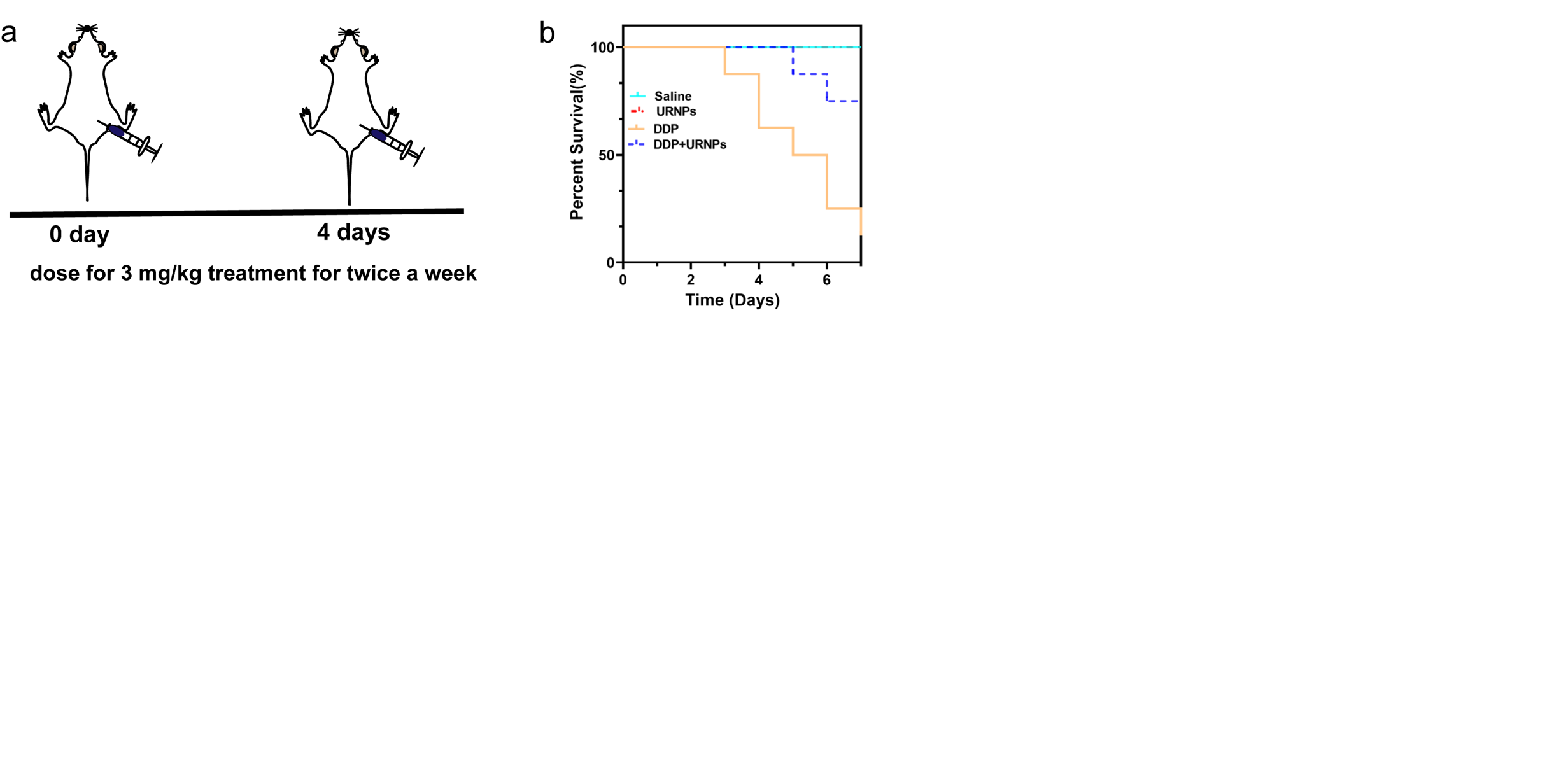


**Figure S27**. The BALB/C mice were divided into four group: Saline, URNPs (3 mg/kg, twice a week), and DDP (3 mg/kg, twice a week) with or without URNPs (3 mg/kg, twice a week) throughout the observation period (*n* = 8) (a)Schematic illustration of mice model. (b)The survival probability of mice treated in different groups.
